# Boosting photoelectrochemical efficiency by near-infrared-active lattice-matched morphological heterojunctions

**DOI:** 10.1038/s41467-021-24569-9

**Published:** 2021-07-14

**Authors:** Guo-Qiang Liu, Yuan Yang, Yi Li, Taotao Zhuang, Xu-Feng Li, Joshua Wicks, Jie Tian, Min-Rui Gao, Jin-Lan Peng, Huan-Xin Ju, Liang Wu, Yun-Xiang Pan, Lu-An Shi, Haiming Zhu, Junfa Zhu, Shu-Hong Yu, Edward H. Sargent

**Affiliations:** 1 Division of Nanomaterials & Chemistry, Hefei National Laboratory for Physical Sciences at the Microscale, Institute of Energy, Hefei Comprehensive National Science Center, CAS Center for Excellence in Nanoscience, Department of Chemistry, Institute of Biomimetic Materials & Chemistry, Anhui Engineering Laboratory of Biomimetic Materials, University of Science and Technology of China, Hefei, China; 2 Department of Electrical and Computer Engineering, University of Toronto, 35 St George Street, Toronto, ON Canada; 3 Department of Chemistry, Zhejiang University, Hangzhou, Zhejiang China; 4 Engineering and Materials Science Experiment Center, University of Science and Technology of China, Hefei, Anhui China; 5 Center for Micro- and Nanoscale Research and Fabrication, University of Science and Technology of China, Hefei, Anhui China; 6 National Synchrotron Radiation Laboratory, University of Science and Technology of China, Hefei, China; 7 School of Chemistry and Chemical Engineering, Hefei University of Technology, Hefei, Anhui China

**Keywords:** Photocatalysis, Light harvesting, Photocatalysis, Devices for energy harvesting, Photocatalysis

## Abstract

Photoelectrochemical catalysis is an attractive way to provide direct hydrogen production from solar energy. However, solar conversion efficiencies are hindered by the fact that light harvesting has so far been of limited efficiency in the near-infrared region as compared to that in the visible and ultraviolet regions. Here we introduce near-infrared-active photoanodes that feature lattice-matched morphological hetero-nanostructures, a strategy that improves energy conversion efficiency by increasing light-harvesting spectral range and charge separation efficiency simultaneously. Specifically, we demonstrate a near-infrared-active morphological heterojunction comprised of BiSeTe ternary alloy nanotubes and ultrathin nanosheets. The heterojunction’s hierarchical nanostructure separates charges at the lattice-matched interface of the two morphological components, preventing further carrier recombination. As a result, the photoanodes achieve an incident photon-to-current conversion efficiency of 36% at 800 nm in an electrolyte solution containing hole scavengers without a co-catalyst.

## Abstract (web summary)

The solar conversion efficiencies of photoelectrochemical catalysis are hindered by the light harvesting range. Here, the authors use near-infrared-active photoanodes that feature lattice-matched morphological hetero-nanostructures to realize efficient photoelectrochemical hydrogen production.

## Introduction

The direct conversion of solar energy into chemical fuels offers a means to store renewable energy^1–8^. However, the practical application of photoelectrochemical (PEC) hydrogen production is impeded today by its low energy conversion efficiency^9–13^.

The primary obstacle limiting hole-involved photoelectrocatalytic reactions is the low concentration of photoholes (*C*_*h+*_) that reach the surface of catalysts^14–16^. Improving the number of available photons to increase *C*_*h+*_ represents an important avenue to achieving high-efficiency photoanodes.

A wide range of semiconductors^17–21^ have shown application as photoanode materials; however, their large bandgaps limit light absorption to the ultraviolet (UV, below 400 nm) and visible (vis, 400 to 700 nm) wavelengths. Extending optical absorption into the infrared region (IR, above 700 nm) enables further utilization of the remaining 50% of solar photon fluence and will promote these devices in toward the Shockley–Queisser (SQ) efficiency limit^17,22^. Upconverting phosphors^23,24^ and metal plasmonic nanostructures^25^ have previously been integrated into near-infrared (NIR) photoelectrodes (Supplementary Table 1). Unfortunately, the low efficiency of upconversion luminescence and the short sub-picosecond lifetime of hot charge carriers lead to impractical photocurrent densities on the order of microamperes per square centimeter^26–28^.

Narrow bandgap (NBG) semiconductors^18^ that exhibit a wide NIR absorption range, a large absorption cross section, and long-lived charge carriers, offer a promising avenue toward NIR-active photoelectrodes that utilize band-edge carriers. However, electron–phonon interactions and non-radiative recombination at defects have, until now, resulted in short-lived photoexcited carriers in these NBG semiconductors, a fact that prevents the needed surface redox reactions from proceeding efficiently^27^. Consequently, NIR absorber-based photoanodes have been limited to an incident photon-to-current conversion efficiency (IPCE) below 3%^18,29–31^.

With these considerations in mind, we focused on the development of NIR-active morphological heterostructures (MHs) with lattice-matched interfaces as a means to enhance the energy conversion efficiency of NBG semiconductors. Lattice-matched morphological heterojunctions reduce carrier-trapping interfacial defects that generally exist at interfaces of two distinct semiconductors with large lattice mismatch^32,33^. When the morphological heterojunction is selected for a broadband absorption spectral range, and when it provides a low recombination rate, it offers avenues to increased energy conversion efficiencies.

Here, we report an efficient NIR-active photoanode by engineering a BiSeTe ternary alloy in the form of lattice-matched morphological heterojunctions (BST-MHs). We grow epitaxially thin nanosheets onto nanotubes that absorb NIR radiation up to 1100 nm. We verify the existence of a lattice-matched type-II heterojunction and the ultrafast charge transfer process responsible for enhancing electron-hole separation. The resulting photoanodes exhibit an IPCE that reaches 36% at 800 nm in an electrolyte solution containing hole scavengers without a cocatalyst.

## Results

### BST-MH synthesis and characterization

Bismuth chalcogenides (Bi_2_Te_3_ and Bi_2_Se_3_) with bulk bandgaps of 0.15–0.35 eV and optical absorption coefficients, *α*, on the order of 10^5^ cm^−1^ hold potential as NIR absorption materials^34–36^. However, such NBGs usually lead to fast electron-hole recombination under solar irradiation, thus limiting the solar conversion efficiency^27^.

Fortunately, the quantum confinement of few-layer bismuth chalcogenides with thicknesses below their exciton Bohr radii (52.5 nm for Bi_2_Se_3_) enables them layer-dependent carrier mobilities (on the order of 10^3^ to 10^4^ cm^2^ V^−1^ s^−1^) and tailored bandgaps^37,38^. We hypothesized that building lattice-matched type-II morphological heterojunctions based on quantum-confined ternary BiSeTe semiconductor materials with suitable bandgaps could enable engineering photoanodes toward more efficient NIR-driven solar energy conversion.

**Fig. 1:**
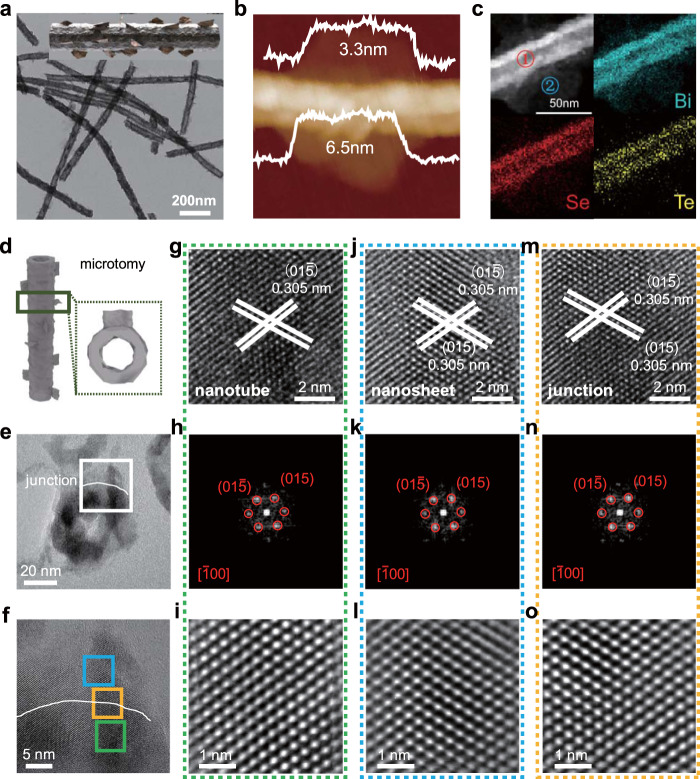
Morphological and compositional characterization of BST-MHs. **a** TEM image of BST-MHs. Inset is a schematic geometric model. **b** AFM image of BST-MHs. **c** EDS mapping images of BST-MHs. **d** Schematic of the sliced BST-MHs. **e**, **f** TEM image and HRTEM image of the junction. **g**–**i** HRTEM image, FFT, and the inverse FFT of the nanotube in the green domain of (**f**). **j**–**l** HRTEM image, FFT, and the inverse FFT of the nanosheet in the blue domain of (**f**). **m**–**o** HRTEM image, FFT, and the inverse FFT of junction in the yellow domain of (**f**).

We designed BiSeTe morphological heterojunctions (BST-MHs) using a strategy that combines chemical transformation with epitaxial growth. The BST-MHs—constructed in the form that few-layer nanosheets epitaxially grow on nanotubes—were chemically transformed from templates of Te_x_Se_y_@Se core-shell nanowires (Supplementary Fig. 1, see Methods for further synthetic details)^39,40^. We examined the BST-MHs using low-magnification transmission electron microscopy (TEM, Fig. 1a): these revealed hierarchical nanostructures with ultrathin nanosheets grown around the nanotubes. We further confirmed the ultrathin nature of the nanosheets via atomic force microscopy (AFM, Fig. 1b). The hierarchical nanostructure is expected to enable both rapid carrier transport to improve the charge separation, and also a high surface-to-volume ratio to facilitate redox reactions.

The peaks in the powder X-ray diffraction (XRD) pattern of BST-MHs (Supplementary Fig. 2a) were indexed to the rhombohedra-phase Bi_2_Se_3_ (JCPDS card No. 33-0214, space group of R-3m) with an overall shift to lower 2*ϴ* values, a finding we attribute to the incorporation of tellerium^39^. Elemental mapping images of the BST-MHs (Fig. 1c) and energy dispersive spectra (EDS) (Supplementary Fig. 2b–d) revealed that the Bi and Se elements are homogeneously distributed in the nanotubes and nanosheets; while Te is mainly distributed in the nanotubes, consistent with the core-shell structure of the Te_x_Se_y_@Se nanowire templates.

High-resolution transmission electron microscopy (HRTEM) images of the BiSeTe nanotubes and nanosheets (Supplementary Figs. 3 and 4) show lattice spacings of 0.207 and 0.305 nm, which we index to the (110) and (015) planes of the rhombohedral BiSeTe ternary compounds, respectively. Each corresponding fast Fourier transform (FFT) image exhibits the diffraction pattern associated with rhombohedral BiSeTe, matching well with XRD patterns.

We further carried out HRTEM to investigate lattice registration at the nanotube/nanosheet interface. Considering that ultrathin nanosheets randomly grow around the nanotubes in a hierarchical manner, we cross-sectioned the unidirectionally aligned nanowires to observe them along different directions. HRTEM images on the nanotubes, nanosheets, and tube/sheet interfaces show lattice spacing of 0.305 nm, which we index to the (015) plane of BiSeTe ternary compounds (Fig. 1d–o). FFTs obtained from these HRTEM images indicate a single diffraction pattern assigned to the [$\bar{1}00$] crystal zone axis of BiSeTe. Similar results can also be found in Supplementary Figs. 5–7. We propose that the lattice-matched epitaxial growth reduces detrimental defects at the interface, which would contribute to efficient charge separation.

### Energy band alignment of BST-MHs

Reducing the thickness of the BiSeTe ternary alloy down to a quantum-confined size is expected to change the energy band structure and corresponding optical properties, and avoid strong electron-phonon scattering^41–43^. To investigate the band alignment of BST-MHs in detail, we tuned the shape of the ternary alloy BiSeTe material to follow only a nanotube (BST-NT) or a nanosheet (BST-NS) structure while retaining the same components and crystal structures for comparison (Supplementary Fig. 8). HRTEM images, EDS spectra (Supplementary Fig. 9), and XPS spectra (Supplementary Fig. 10) indicated that the plain BST-NTs and BST-NSs have the crystal orientations, grain boundaries, and elemental compositions of their counterparts in BST-MHs.

**Fig. 2:**
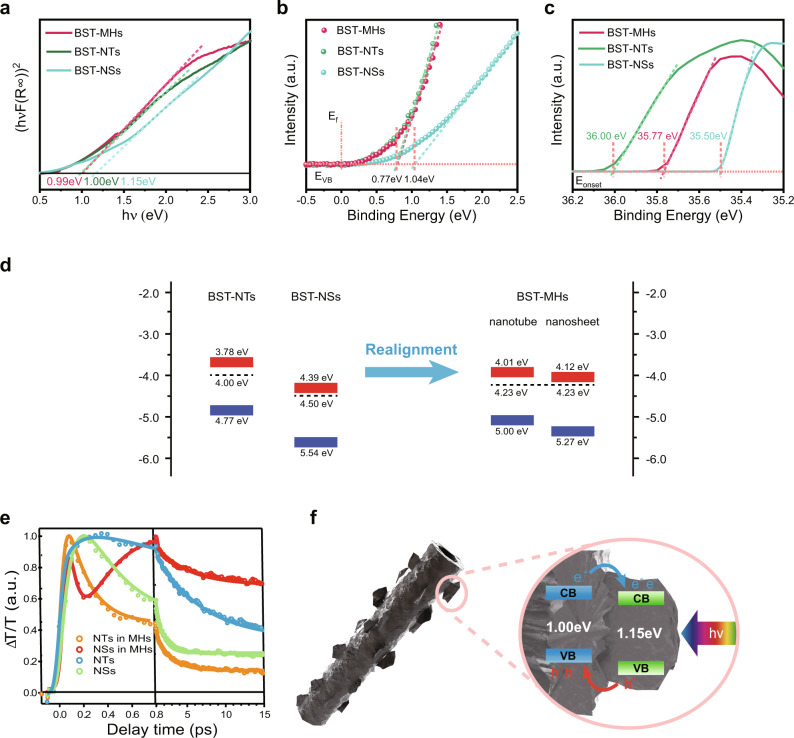
Spectroscopic characterization and schematic energy band diagrams. **a** Tauc plots of BST-MHs, BST-NTs, and BST-NSs derived from UV-Vis diffuse reflectance spectra. **b** UPS spectra of BST-NTs, BST-NSs, and BST-MHs, showing their relative energies between valence band maximum and Fermi level. **c** Onset level (*E*_onset_) of the UPS spectra for BST-MHs, BST-NTs, and BST-NSs, respectively. **d** Energy band diagrams of BST-NTs, BST-NSs, and BST-MHs. **e** Corresponding TA kinetics at B_1_ and B_2_ of BST-NTs, BST-NSs, and BST-MHs. The circles are experimental data and the solid lines are their exponential fits. **f** Schematic of charge-separation processes in BST-MHs under solar irradiation.

To verify their optical bandgaps (*E*_g_), we transformed the UV-vis diffuse reflectance spectra into a Tauc plot (Fig. 2a). The optical bandgaps of BST-MHs, BST-NTs, and BST-NSs are 0.99, 1.00, and 1.15 eV, respectively. In contrast to the bandgaps of bulk Bi_2_Se_3_ (0.35 eV) and bulk Bi_2_Te_3_ (0.15 eV), the BST-MH samples show larger optical bandgaps, a finding we assign to the quantum confinement effect^37,39,43–45^. We further characterized their band structures by ultraviolet photoelectron spectroscopy (UPS). Figure 2b shows the relative electron binding energies between the valence band maximum and Fermi level (*E*_VBM_-*E*_f_), which are −0.77, −0.77, and −1.04 eV, for BST-MHs, BST-NTs, and BST-NSs. Meanwhile, we calculated, using the equation *Φ* = *hν* − *E*_onset_, that the work functions (*Φ*, the relative energy between the Fermi level and the vacuum level) of BST-MHs, BST-NTs, and BST-NSs are 4.23, 4.00, and 4.50 eV, respectively (Fig. 2c).

Considering the relationship between the *E*_g_, *E*_VBM_ – *E*_f_, and *Φ*, we, therefore, present the energy band diagrams before and after alignment (Fig. 2d). The *E*_f_ difference between plain BST-NTs and BST-NSs drives electrons in BST-NTs to flow into BST-NSs after the formation of heterojunctions, resulting in Fermi level alignment at 4.23 eV. In this way, the type-II band structure formed in the BST-MHs facilitates charge transfer and separation. Under solar irradiation, photoexcited electrons in BST-NTs flow into BST-NSs, while photoexcited holes can transfer from BST-NSs into BST-NTs.

To verify the proposed band alignment and charge transfer mechanism in the BST-MHs, we further carried out femtosecond transient absorption (TA) spectroscopy on BST-NTs, BST-NSs, and BST-MHs. After photoexcitation, BST-NTs and BST-NSs instantaneously show bleach peaks centered at ~645 and ~590 nm, respectively (Supplementary Fig. 11a, b). We attribute the bleach mainly to the state filling of conduction band edges, given the much smaller effective mass of an electron than a hole in these materials. Thus, these bleach kinetics (Fig. 2e) inform the electron population^38,43^. We extracted a half-lifetime of ~7 and ~2 ps from TA kinetics of BST-NTs and BST-NSs (Supplementary Table 2).

In BST-MHs, the bleach peak from the NTs at ~645 nm appears instantaneously after photoexcitation, but decays quickly within ~0.4 ps. This process is accompanied by the rising bleach peak at ~590 nm associated with the NSs in the same timescale (Supplementary Fig. 11c), indicating an ultrafast electron transfer process from the NT domain to the NS domain in BST-MHs driven by the type-II band alignment (Fig. 2f). The resulting electrons in the NS domain of BST-MHs have a half-lifetime of ~80 ps (Supplementary Fig. 11d), which is more than one order of magnitude longer than that in BST-NTs and BST-NSs. The much longer electron lifetime in MHs is attributed to spatial electron-hole separation in such type-II morphological heterojunctions.

### Photoelectrochemical hydrogen production performance

We implemented the PEC H_2_ production half-reaction to test NIR-MHs for promise in performance. We prepared thickness-controlled photoanodes and compared their PEC performance using Na_2_SO_3_/Na_2_S as hole scavengers (Supplementary Figs. 12–14)^46–48^. The optimal thicknesses for BST-MHs, NTs, NSs, and NTs+NSs are determined to be 3, 2, 3, and 2 layers, respectively (Supplementary Fig. 15a–d).

**Fig. 3:**
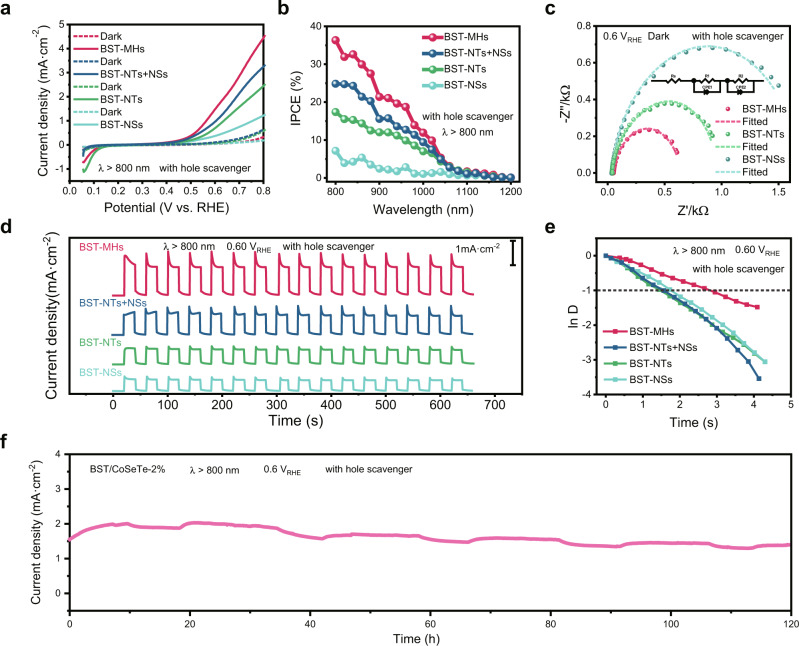
PEC performance. **a**, **b** Current-potential curves (**a**), IPCEs (**b**) of the BST-MH, BST-NT, BST-NS, and BST-NT + BST-NS photoanodes under NIR light irradiation (*λ* > 800 nm, 100 mW cm^−2^). **c** Electrochemical impedance spectra measured for different photoanodes with hole scavengers, *Z'*’ and *Z''* are the real and imaginary parts of impedance. The inset shows the equivalent circuit. **d**, **e** Transient current-time curves (**d**) and normalized plots of photocurrent density-time dependence (**e**) of BST-MH, BST-NT, BST-NS, and BST-NT + BST-NS photoanodes at 0.60 V_RHE_ under NIR light irradiation (*λ* > 800 nm, 100 mW cm^−2^). **f** Stability test of the BST/CoSeTe-2% photoanode at 0.60 V_RHE_ under NIR light irradiation (*λ* > 800 nm, 100 mW cm^*−*2^). All experiments were measured in an electrolyte solution containing 0.08 mol L^−1^ Na_2_S and 0.50 mol L^−1^ Na_2_SO_3_ as hole scavengers (pH = 12.9).

As shown in Fig. 3a, the anodic photocurrent density of BST-MHs sharply increased at potentials above 0.5 V_RHE_, reaching 3.7 mA cm^−2^ at 0.75 V_RHE_. In stark contrast, photocurrent densities for BST-NTs and BST-NSs were 1.98 and 0.96 mA cm^−2^ at 0.75 V_RHE_. The BST-MHs achieve a twofold higher photocurrent density compared with the best previous reports (Supplementary Table 3). Compared to BST-NT and BST-NS photoanodes, BST-NT + NS photoanodes show higher photocurrent due to the hierarchical structure formed by a simple stacking of nanosheets and nanotubes, which improves the absorption capacity of the photoelectrode by light scattering (Supplementary Fig. 12)^5^. However, the BST-MHs showed a larger photocurrent than that of the individual BST-NTs, BST-NSs, and the BST-NT + NSs. We attribute the enhanced performance of the BST-MHs to lattice-matched type-II morphological heterojunctions, which are conducive to the separation and transport of charge carriers. Figure 3b shows that the IPCE of BST-MHs is higher than that of BST-NTs, BST-NSs, and the mixture in the range of 800–1100 nm. The IPCE of the BST-MH photoanode reached 36% at 800 nm in an electrolyte solution containing hole scavengers with an applied potential of 0.6 V_RHE_, demonstrating a threefold enhancement compared to the existing record^18^. In addition, the IPCE values of these photoanodes approached 0% at 1200 nm, in accordance with their optical bandgaps. The high photocurrent density and IPCE can be attributed to charge separation with long-lived charge carriers driven by type-II band alignment, consistent with TA analysis and electrochemical impedance spectra (Fig. 3c and Supplementary Table 4). These results support the view that NIR-MHs composed of an NBG semiconductor serves as a NIR-active photoanode material.

The transient photocurrent response of the BST-MH photoanode was acquired at a constant potential of 0.60 V_RHE_ with a light chopper under NIR light (Fig. 3d). Results show that the stable photocurrent density of BST-MHs (1.1 mA cm^−2^) is significantly enhanced compared with other photoanodes. We further analyzed the photocurrent decay at 0.6 V_RHE_. In contrast to other photoanodes investigated in this work, the transient decay time, *τ*, of the BST-MH photoanode is the longest, indicating that the lattice-matched MHs suppress carrier recombination and accelerate carrier migration (Fig. 3e). The Mott–Schottky plots reveal that the flat-band potential of the n-type BST-MHs is 0.425 V_RHE_. We estimated the carrier concentration in the BST-MHs using the double-layer capacitance^49^, which was on the order of 10^20^ cm^−3^ (Supplementary Fig. 15e). We also measured its open-circuit photovoltage under different solar sources to estimate the attainable photovoltage under PEC operating conditions (Supplementary Fig. 15f). In addition, to achieve unassisted solar water splitting, we aim to build the next generation electrode structures to increase the photovoltage. These electrodes can be coupled with high-efficiency tandem PV-PEC cells to achieve full utilization of the solar spectrum, thus improving the energy conversion efficiency of the device^6,7^.

To mitigate the spike in transient current and photocorrosion of the photoanode—a consequence of the inefficient hole-involved sulfite oxidation reaction—we introduced a cobalt-based cocatalyst (CoSeTe) to the BST-MHs (Supplementary Figs. 16–18). We tested the transient photocurrent response and the stability of the photoanode for PEC hydrogen production in electrolytes containing Na_2_S and Na_2_SO_3_ (Fig. 3f and Supplementary Figs. 19, 20). Under NIR light, the transient decay time *τ* of the BST/CoSeTe-2% photoanode is extended threefold, and the photoanode remained stable during a 120 h experiment. In contrast to plain BST-MHs, the BST/CoSeTe photoanode maintained its morphology after 120 h (Supplementary Fig. 20d, e). This demonstrates that the separation and consumption of carriers in the photoanode is accelerated after loading the cocatalyst, avoiding photocorrosion, and thereby ensuring its stability.

To challenge further the efficiency of carrier separation and application-relevance of MHs, we conducted PEC water splitting under NIR light. As shown in Supplementary Fig. 21, the lower onset potential and higher photocurrent revealed that the lattice-matched type-II MHs efficiently enhanced the separation and transport of photogenerated carriers. After loading a cocatalyst to accelerate water oxidation kinetics, the BST/CoSeTe-2% photoanode showed the lowest onset potential and highest photocurrent. The BST/CoSeTe-2% photoanode showed improved stability compared to the BST-MH photoanode. We attribute the moderate photocurrents to the fact that a more suitable cocatalyst to promote the water oxidation reaction has yet to be found. With the identification of suitable cocatalysts as a clear next step, these BiSeTe-based semiconductors provide a route to more efficient and stable PEC water splitting.

In addition, we also tested the PEC performance of the BST-MH photoanode under visible light illumination (*λ* > 420 nm), as displayed in Supplementary Fig. 22. A significant quantity of H_2_ bubbles was observed on the Pt counter electrode (Supplementary movie 1). The anodic photocurrent density of BST-MHs reached 7.1 mA cm^−2^ at 0.75 V_RHE_. Since sulfite oxidation is thermodynamically and kinetically more facile, BST-MH photoanodes have a lower observed overpotential, which results in the low onset potential (0.2 V_RHE_) of the anode photocurrent^12^. The IPCE of the BST-MH photoanode reached 73% at 400 nm in an electrolyte solution containing hole scavengers with an applied potential of 0.6 V_RHE_.

Further to prove the advantage of MHs, we grew two types of lattice-mismatched hetero-nanostructures (BST/Bi_2_S_3_ and BST/MoS_2_). These had a significantly higher density of interface defects due to the larger difference in lattice spacing and crystalline orientation at the interface (Supplementary Figs. 23–28, see Methods for synthetic details). The poor PEC performance of BST/Bi_2_S_3_ and BST/MoS_2_ agrees with the picture that reducing interfacial defects is important to improve charge separation (Supplementary Fig. 29).

**Fig. 4:**
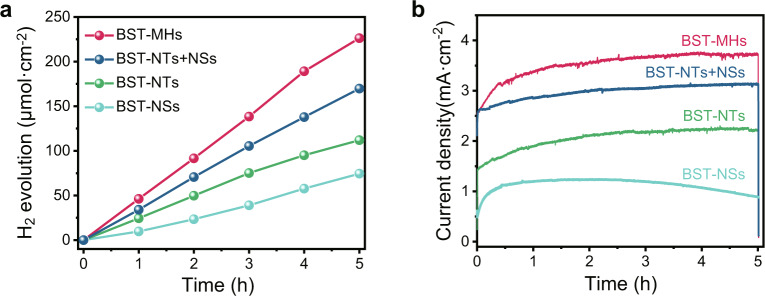
PEC H_2_ evolution. **a**, **b** H_2_ evolution (**a**) and photocurrent densities (**b**), of the BST-MH, BST-NT, BST-NS, and BST-NT + BST-NS photoanodes at 0.6 V_RHE_. The experiments were measured in an electrolyte solution containing 0.08 mol L^−1^ Na_2_S and 0.50 mol L^−1^ Na_2_SO_3_ as hole scavengers (pH = 12.9) under NIR light irradiation (*λ* > 800 nm, 100 mW cm^−2^).

We further quantified the H_2_ evolution rate during the PEC H_2_ production reaction under NIR light. The BST-MHs achieved high performance of more than 46 μmol cm^−3^ h^−1^ at 0.60 V_RHE_ under NIR light (Fig. 4a). In contrast, the H_2_ production rates for BST-NTs, BST-NSs, and the mixture of the two were 22, 15, and 34 μmol cm^−3^ h^−1^, respectively. The photocurrent densities showed a slight decay after a continuous 5 h measurement, which was attributed to the hole-accumulation-induced photocorrosion (Fig. 4b). The H_2_ evolution rate of the BST-MH photoanode reached 85 μmol cm^−3^ h^−1^ at 0.60 V_RHE_ under visible light illumination (>420 nm) (Supplementary Fig. 30a). Meanwhile, the morphology and crystal structure showed no observable changes (Supplementary Fig. 30b, c). XPS spectra (Supplementary Fig. 30d–f) indicated that the photoanode surface was slightly oxidized after the long-term reactions, which is in accordance with the modest performance degradation.

## Discussion

In summary, we have established NIR-MHs as a class of new photoanode materials, which possess a wide light-harvesting spectral range and efficient charge separation. In delving deeper, the BST-MHs constructed by few-layer nanosheets grown on nanotubes showed a high absorption of NIR light (*λ* > 800 nm) and a high photoinduced carrier separation due to the lattice-matched morphological heterojunctions. The BST-MH photoanode showed an IPCE of 36% at 800 nm in an electrolyte solution containing hole scavengers and a photocurrent density of 1.5 mA cm^−2^ at 0.60 V_RHE_ under NIR light. When we introduce CoSeTe, the BST/CoSeTe-2% photoanode produces hydrogen under NIR light for 120 h. This study offers new possibilities to engineer efficient NIR-active PEC devices by integrating the advantages of NBG semiconductors into a lattice-matched morphological heterojunction configuration.

## Methods

### Chemicals

Na_2_TeO_3_ (98%) was purchased from Maya Reagent (China). Polyvinylpyrrolidone (PVP, K30), Se powder (99%), hydrazine hydrate (85%, w/w%), aqueous ammonium solution (25–28%, w/w%), acetone, ethylene glycol (EG), thiourea, Bi(NO_3_)_3_·5H_2_O, Na_2_SeO_3_, Na_2_TeO_3_, KOH, (NH_4_)_6_Mo_7_O_24_·4H_2_O, *N*, *N*-dimethylformamide (DMF), trichloromethane (CHCl_3_), sodium sulfide (Na_2_S), and sodium sulfite (Na_2_SO_3_) were purchased from the Shanghai Reagent Company (P. R. China). All chemicals were used as received without further purification.

### Synthesis of Te nanowires

In a typical synthesis^40^, 10.0 g PVP, 0.922 g Na_2_TeO_3_, and 320 mL double-distilled deionized water (DIW, 18.23 MΩ) were added into a 500 mL Teflon-lined stainless steel autoclave under vigorous magnetic stirring. Then, 33.5 mL aqueous ammonium solution and 16.5 mL hydrazine hydrate were added to the solution in the autoclave. After 30 min, the autoclave was maintained at 180 °C for 3 h and then allowed to cool to room temperature naturally.

### Synthesis of Te_x_Se_y_@Se nanowires

In a typical synthesis^39^, Te_x_Se_y_@Se nanowires with different Te:Se molar ratios can be obtained using Te nanowires as templates and controlling the Te:Se ratio by varying the amount of Se-hydrazine solution. In order to synthesize Te_x_Se_y_@Se nanowires with a Te:Se ratio of 1:8, 0.4 mmol of the Te nanowire solution was mixed with 100 mL acetone to extract the Te nanowires. Then, the Te nanowires were redispersed in 70 mL DIW to form a homogenous solution. After that, a certain amount of Se-hydrazine solution (3.2 mmol Se powder dissolved in 1.0 mL hydrazine hydrate) was dropped slowly into the previous solution and DIW was added to maintain a total final volume of 80 mL. The final solution was heated at 40 °C for 12 h under magnetic stirring. Finally, the solution was aged at 80 °C for another 12 h.

### Synthesis of BiSeTe ternary alloy lattice-matched morphological heterojunctions

In order to prepare the BiSeTe ([Te]:[Se] = 1:8) ternary alloy lattice-matched morphological heterojunctions (BST-MHs), 0.6 mmol of Bi(NO_3_)_3_·5H_2_O, 38 mL of DIW, and 2 mL of hydrazine hydrate, 0.9 mmol of Te_x_Se_y_@Se nanowires solution ([Te]:[Se] = 1:8) were mixed in a 100 mL Teflon-lined stainless steel autoclave under magnetic stirring. Then the autoclave was sealed and maintained at 160 °C for 12 h. After the reaction, the final products were collected by centrifugation (11,000 x*g*, 3 min) and washed three times with ethanol.

### Synthesis of BiSeTe ternary alloy nanotubes

About 0.9 mmol of Te_x_Se_y_@Se nanowires ([Te]:[Se] = 1:8) solution were collected by centrifugation (17,000 x*g*, 5 min) to extract the Te_x_Se_y_@Se nanowires. Then the Te_x_Se_y_@Se nanowires were redispersed in 78 mL EG to form a homogenous solution. After that, 0.6 mmol of Bi(NO_3_)_3_·5H_2_O, 2 mL of hydrazine hydrate, and Te_x_Se_y_@Se nanowire solution were mixed in a 100 mL Teflon-lined stainless steel autoclave under magnetic stirring. Then the autoclave was sealed and maintained at 160 °C for 12 h. After the reaction, the final products were collected by centrifugation (11,000 x*g*, 3 min) and washed three times with ethanol.

### Synthesis of BiSeTe ternary alloy nanosheets

In the synthesis of BST-NSs, 0.4 mmol of Bi(NO_3_)_3_·5H_2_O, 0.533 mmol Na_2_SeO_3_, 0.067 mmol Na_2_TeO_3_, 1.0 g PVP, and 1.0 g KOH were dissolved in 80 mL EG and heated to 200 °C for 24 h. After the reaction, the final products were collected by centrifugation (4200 x*g*, 3 min) and washed three times with ethanol.

### Synthesis of BiSeTe/Bi_2_S_3_

About 0.3 mmol of Bi(NO_3_)_3_·5H_2_O, 40 mL of DIW, 30 mL of EG, and 0.3 g of thiourea, 10 mL of BST-NTs solution were mixed in a 100 mL Teflon-lined stainless steel autoclave under magnetic stirring. Then the autoclave was sealed and maintained at 110 °C for 12 h. After the reaction, the final products were collected by centrifugation (11,000 x*g*, 3 min) and washed three times with ethanol.

### Synthesis of BiSeTe/MoS_2_

A total of, 20 mL BST-NTs solution was collected by centrifugation (11,000 x*g*, 5 min) to extract the BST-NTs. Then the BST-NTs were re-dispersed in 80 mL DIW to form a homogenous solution. After that, 0.15 mmol of (NH_4_)_6_Mo_7_O_24_·4H_2_O and 4.5 mmol of thiourea were mixed in a 100 mL Teflon-lined stainless steel autoclave under magnetic stirring. Then the autoclave was sealed and maintained at 180 °C for 12 h. After the reaction, the final products were collected by centrifugation (11,000 x*g*, 3 min) and washed three times with ethanol.

### Synthesis of BiSeTe/CoSeTe

We chose BiSeTe/CoSeTe-1% to demonstrate the synthesis. About 0.594 mmol of Bi(NO_3_)_3_·5H_2_O, 0.009 mmol of Co(NO_3_)_2_·6H_2_O, 38 mL of DIW, 2 mL of hydrazine hydrate, and 0.9 mmol of Te_x_Se_y_@Se nanowires solution ([Te]:[Se] = 1:8) were mixed in a 100 mL Teflon-lined stainless steel autoclave under magnetic stirring. The autoclave was sealed and maintained at 160 °C for 12 h. After the reaction, the final products were collected by centrifugation (11,000 x*g*, 3 min) and washed three times with ethanol.

### Photoanode fabrication

The photoanodes were fabricated by a Langmuir–Blodgett technology (Nima Technology, 312D) with DIW as a subphase^50^. The prepared BST-MH solution (1 mL) was centrifuged after adding alcohol (3 mL) and then the BST-MHs were re-dispersed into a stirred solution of chloroform (1 mL) and DMF (1 mL) to form a homogeneous solution. The final solution was dispensed from a 100 μL syringe onto the water subphase dropwise to form a film. After 10 min, the film was compressed by a Wilhelmy plate with a compression rate of 20 cm^2^ min^−1^. The final film was lifted with an FTO glass (1 cm × 3 cm). By repeating the lifting process, the thickness of BST-MH film on the FTO glass can be modulated.

### Femtosecond transient absorption (TA) measurement

We used BST-MH, BST-NT, and BST-NS films for TA measurement. For the TA measurement (Time-Tech Spectra, TA100), the fundamental output from Yb:KGW laser (1030 nm, 100 kHz, 220 fs Gaussian fit, Light Conversion Ltd) was separated into two light beams. One beam was introduced to the optical parametric amplifier (ORPHEUS-N, Light Conversion Ltd) to produce a 460 nm pump beam, and the other was focused onto a YAG plate to produce a white light continuum probe beam. A linear array detector was used to collect the transmitted probe light from the sample.

### Photoelectrochemical current measurement

The PEC H_2_ production performance of the photoanodes was tested on a CHI760D electrochemistry workstation in a typical three-electrode configuration. The counter electrode was a Pt foil, and the reference electrode was an Ag/AgCl electrode. The simulated solar illumination was supplied by a 300 W xenon lamp equipped with a NIR filter (*λ* > 800 nm) or a UV light filter (*λ* > 420 nm). The intensity of the incident light was calibrated to 100 mW cm^−2^ with a power meter. Photocurrent measurements were measured in an electrolyte solution containing 0.08 mol L^−1^ Na_2_S and 0.50 mol L^−1^ Na_2_SO_3_ as hole scavengers (pH = 12.9). The electrolyte was thoroughly deaerated by purging with nitrogen before measurements. The photocurrents were collected at a range of −0.9 to −0.15 V vs Ag/AgCl with a scan rate of 10 mV s^−1^. In order to transform the potential vs Ag/AgCl to reversible hydrogen electrode (RHE), the following equation was used^51,52^:

1 $${{E}_{\text{RHE}} = {E}_{\text{Ag}/\text{AgCl}}\,\text{+}\,0\text{.}059\text{pH}}\,+\,{E}_{\text{Ag}/\text{AgCl}}^{^\circ }$$

The ${E}_{\text{Ag}/\text{AgCl}}^{^\circ }$ (3 M KCl) = 0.197 at 25 °C.

### Photoelectrochemical H_2_ evolution measurement

Photoelectrochemical H_2_ production was measured in a PEC cell system connected to a glass-closed gas circulation system. For each measurement, the BST-MH photoanode was immersed in an electrolyte (200 mL) containing Na_2_SO_3_ (0.5 M) and Na_2_S (0.08 M) as hole scavengers. The electrolyte was evacuated three times to completely remove air. The test was conducted under a 300 W xenon lamp equipped with a NIR filter (*λ* > 800 nm, 100 mW cm^−2^) or a UV light filter (*λ* > 420 nm, 100 mW cm^−2^). Using Ar as a carrier gas, the H_2_ evolved from the PEC cell system was quantified through a gas chromatograph (Agilent Technologies Corporation) equipped with a thermal conductive detector. The gas products were sampled every hour.

### Mott–Schottky Plot

The Mott–Schottky Plot was collected with a CHI760D electrochemistry workstation through an impedance-capacitance scan technique with an amplitude of 10 mV and different frequencies (0.5, 1, and 1.5). The electrolyte and the photoanodes were the same as that in PEC current measurements. The donor concentration can be calculated by the equation:^52^

2 $$\frac{1}{{C}^{2}}\,=\,\frac{2\left(V-{V}_{\mathrm{f}}-{kT}/e\right)}{e\varepsilon {\varepsilon }_{\circ }\, {N}_{\mathrm{D}}{A}^{2}}$$

where *C* is the capacitance of the photoanode, *e* is the charge of an electron (1.6 × 10^−19^ C), *ɛ* is the dielectric constant of Bi_2_Se_3_^38^, *ɛ*_*o*_ is the vacuum permittivity (8. 854187817 × 10^−12^ F m^−1^), *V* is the applied bias (vs RHE), *V*_f_ is the flat-band potential (vs RHE), *k* is the Boltzmann constant, *N*_D_ is the donor density (cm^−3^), *A* is the surface area of the photoanodes, and *T* is the temperature (K).

### IPCE measurement

IPCE was also measured on the CHI760D electrochemistry workstation and in the same electrolyte using a three-electrode configuration at 0.6 V_RHE_, with solar irradiation from 400 to 1200 nm. The IPCE can be calculated by the equation:^52^

3 $${\rm{IPCE}}( \% )=\frac{1240\times J}{\lambda \times P}\times 100$$

Where the *J* is the photocurrent density (mA cm^−2^), *P* is the light power density (mW cm^−2^) at *λ*, and *λ* is the wavelength of incident light (nm).

### Characterization

The morphology of the nanostructures was investigated by TEM using a Hitachi H-7700 microscope at 100 kV. SEM images were obtained with a field emission scanning electron microanalyzer (Zeiss Supra 40) at an acceleration voltage of 5 kV. XRD patterns were measured on a Rigaku DMax-γA rotation anode x-ray diffractometer equipped with graphite monochromatized Cu–K radiation (*λ* = 1.54178 Å). HRTEM, high-angle annular dark-field scanning transmission electron microscopy (HAADF-STEM) image, and energy dispersive X-ray (EDX) elemental mapping were obtained by JEOL 2100 F, FEI Tecnai G2 F20 S-Twin microscope, and Talos F200X at 200 kV. X-ray photoelectron spectroscopy (XPS) was recorded using an ESCALAB-MKII x-ray photoelectron spectrometer equipped with Mg Ka radiation as an exciting source. Raman spectra were detected by a Renishaw System 2000 spectrometer using the 514.5 nm line of Ar^+^ for excitation. UV-vis-NIR absorption spectra were obtained by a Shimadzu UV3600 spectrometer at room temperature. AFM was performed by a Bruker Dimension Icon. The infrared spectra (IR) was carried out on a NICOLET Fourier transform infrared spectrometer using pressed KBr pellets. Thermal gravimetric analysis (TGA) of the samples was obtained on a Shimadzu TA-50 thermal analyzer (Shimadzu) at a heating rate of 10 °C min^−1^ from room temperature to 600 °C. The thickness of the BST-MHs film was measured on a Bruker DektakXT. The microtomy was finished on Leica Ultra CUT UC7. In addition, UPS was performed at the Catalysis and Surface Science Endstation (BL11U beamline) in the National Synchrotron Radiation Laboratory (NSRL) in Hefei, China. A sample bias of −5 V was applied to observe the secondary electron cutoff with a photon energy of 40.0 eV. The work function is determined by the difference between the photon energy and the binding energy of the secondary cutoff edge. In actual testing, the Au layer is assigned to 0 eV and acts as a reference for *E*_f_. EIS was measured with a CHI760D electrochemical workstation using a three-electrode configuration.

## Supplementary information

Supplementary Information

Description of Additional Supplementary Files

Supplementary Movie 1

## Data Availability

The data that support the findings of this study are available on request from the corresponding authors (M.-R.G., S.-H.Y., or E.H.S.).
