# Supplementary material for: Boosting photoelectrochemical efficiency by near-infrared-active lattice-matched morphological heterojunctions
Source: Nat Commun. 2021 Jul 14;12:4296. doi: 10.1038/s41467-021-24569-9 (PMC8280183; doi:10.1038/s41467-021-24569-9)
Supplement: Supplementary file 1 — Supplementary Information [file 41467_2021_24569_MOESM1_ESM.pdf]

Supplementary Information

# Boosting photoelectrochemical efficiency by near-infrared-active lattice-matched morphological heterojunctions

Liu et al.

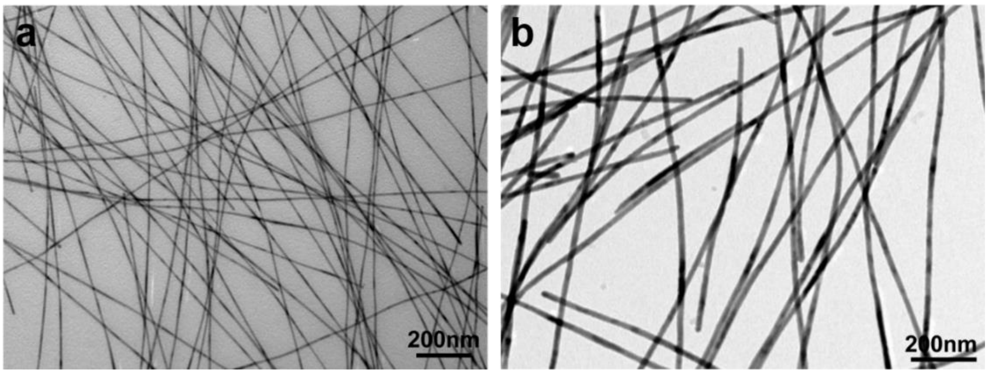


**Supplementary Figure 1 Low-magnification TEM images of Te nanowires and $Te_xSe_y$@Se nanowires. a**, Te nanowires. **b**, $Te_xSe_y$@Se nanowires ([Te]:[Se]= 1:8).

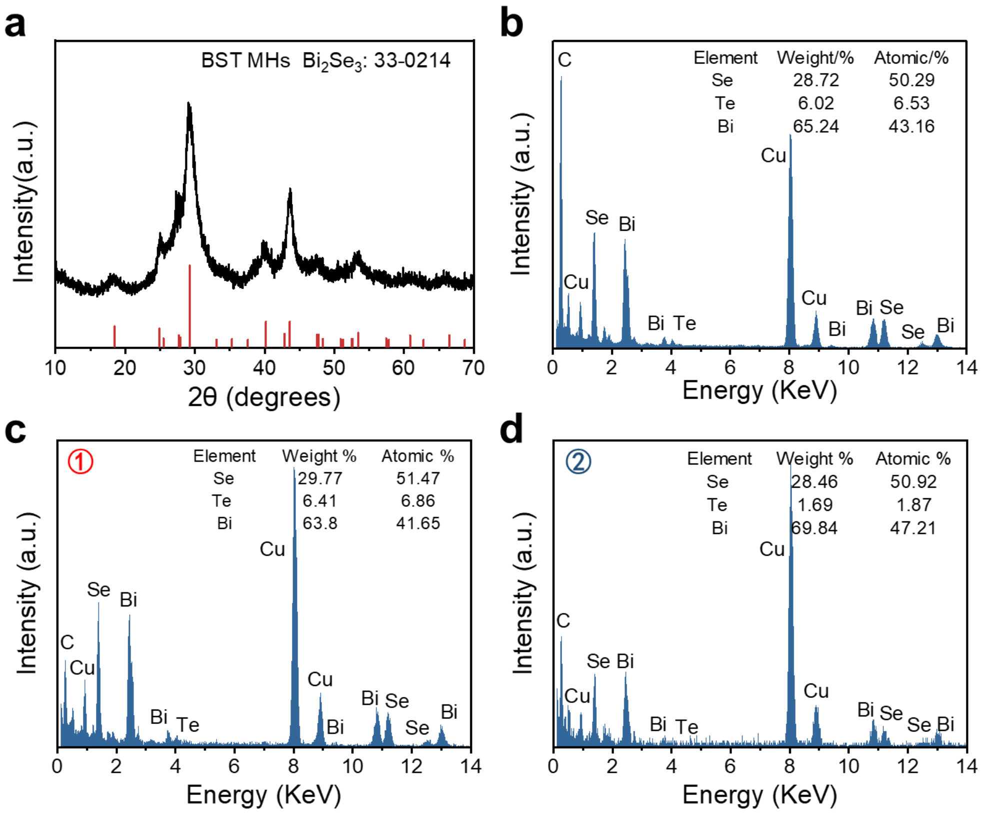


**Supplementary Figure 2 X-ray diffraction patterns and EDS spectra of BST-MHs.** **a**, XRD pattern of BST-MHs. **b**, EDS spectrum of BST-MHs. **c**, **d**, EDS spectra of nanotube and nanosheet components, respectively, of BST-MHs that show the inhomogeneous distribution of Te.

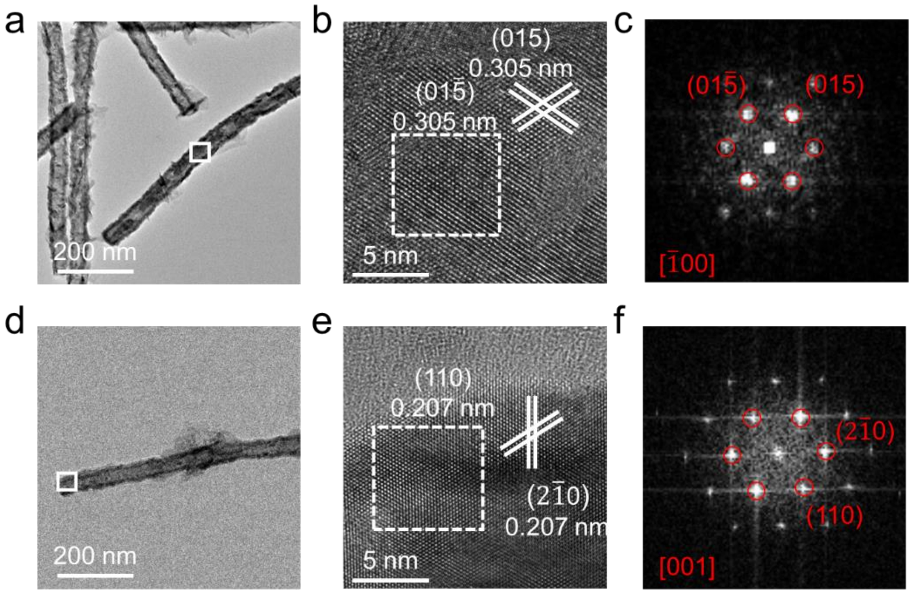


**Supplementary Figure 3 HRTEM and Fast Fourier transform (FFT) images of nanotubes. a**, **d**, TEM images of BST-MHs. **b**, **e**, HRTEM images of nanotubes in the domains highlighted in (**a**) and (**d**). **c, f,** FFTs in the domains highlighted in (**b**) and (**e**).

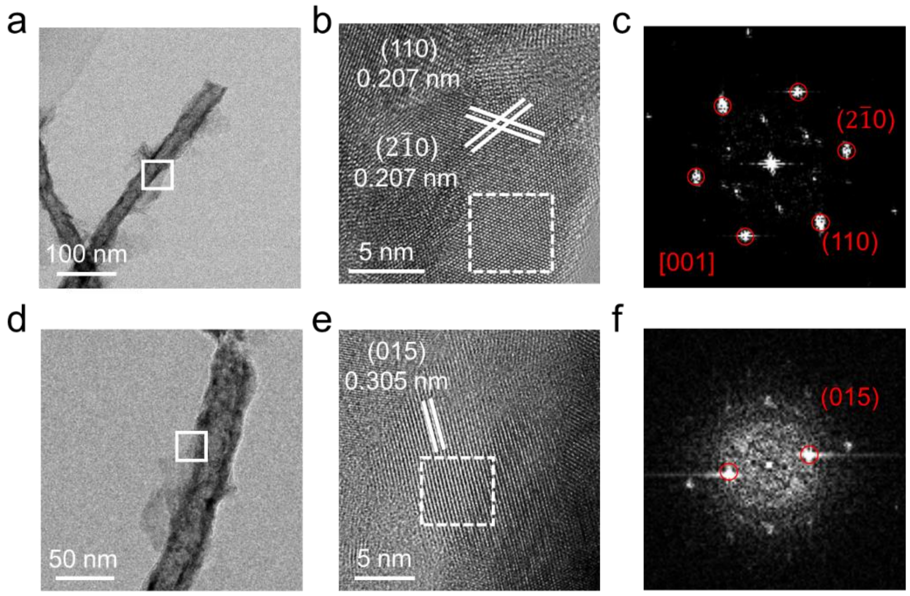


**Supplementary Figure 4 HRTEM and Fast Fourier transform (FFT) images of nanosheets. a**, **d**, TEM images of BST-MHs. **b**, **e**, HRTEM images of nanosheets in the domains highlighted in (**a**) and (**d**). **c**, **f**, FFTs in the domains highlighted in (**b**) and (**e**).

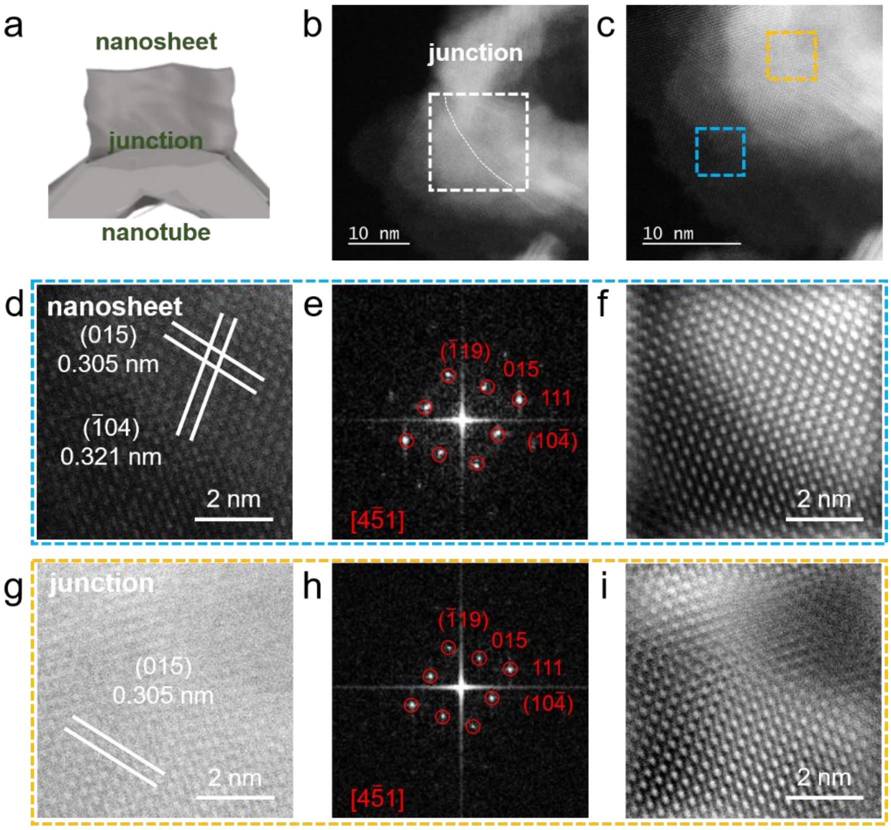


**Supplementary Figure 5 Characterization of the lattice-matched junction between nanotubes and nanosheets.** **a**, Schematic diagram of the sliced sample. **b**, **c**, HR-HAADF-STEM images of the junction. **d**, **e**, **f**, HR-HAADF-STEM image, FFT, and the inverse FFT of nanosheet in the blue domain of (**c**). **g**, **h**, **i**, HR-HAADF-STEM image, FFT, and the inverse FFT of junction in the yellow domain of (**c**).

The HR-HAADF-STEM images of nanotubes, nanosheets, and junctions (Supplementary Fig. 5) show the same lattice spacings of 0.305 nm, which can be indexed to the (015) planes of the rhombohedral BiSeTe ternary alloy, respectively. There is only one electron diffraction pattern in each FFT image obtained from these fixed areas. To better demonstrate the continuity of their crystal lattice, we also performed inversed FFTs with masked spots and these images show good agreement with the HR-HAADF-STEM images.

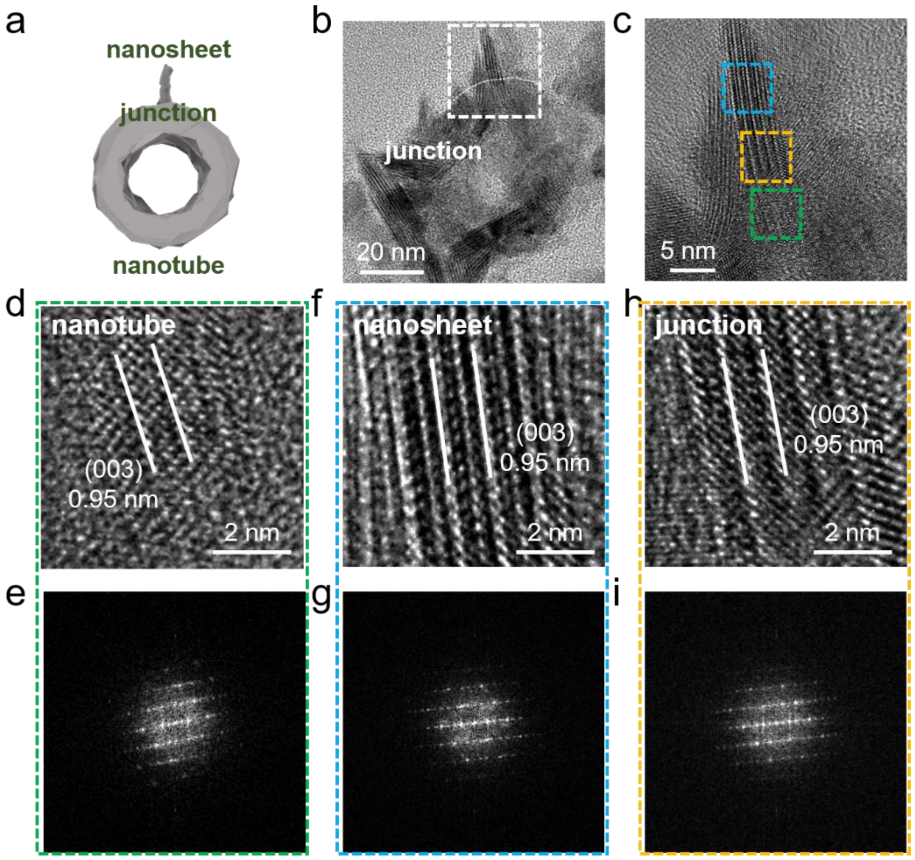


**Supplementary Figure 6 Characterization of the lattice-matched junction between nanotubes and nanosheets. a**, Schematic diagram of the sliced sample. **b**, **c**, TEM and HRTEM images of the junction. **d**, **e**, HRTEM image and FFT of nanotube in the green domain of (**c**). **f**, **g**, HRTEM image and FFT of nanosheet in the blue domain of (**c**). **h**, **i**, HRTEM image and FFT of junction in the yellow domain of (**c**).

Since the nanosheets are randomly and non-directionally distributed on the nanotubes, it is difficult to observe the (003) plane of the nanosheets. However, when the nanosheets are exactly perpendicular to the surface we cut, we can easily observe it. Consistent with our conclusions, the nanosheet, nanotube, and junction have the same lattice spacing of 0.95 nm, which further demonstrates that nanosheets are epitaxially grown on nanotubes. Again, they only have one electron diffraction pattern.

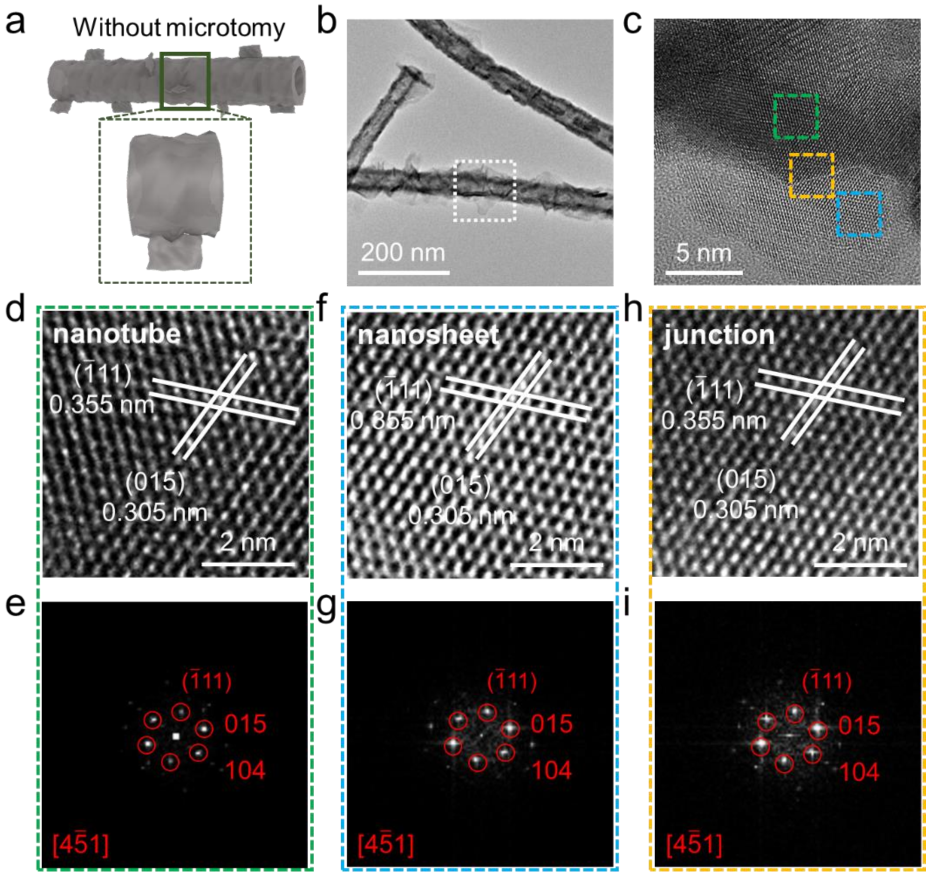


**Supplementary Figure 7 Characterization of the lattice-matched junction between nanotubes and nanosheets.** **a**, Schematic diagram of the junction. **b**, **c**, TEM and HRTEM images of the junction. **d**, **e**, HRTEM image and FFT of nanotube in the green domain of (**c**). **f**, **g**, HRTEM image and FFT of nanosheet in the blue domain of (**c**). **h**, **i**, HRTEM image and FFT of junction in the yellow domain of (**c**).

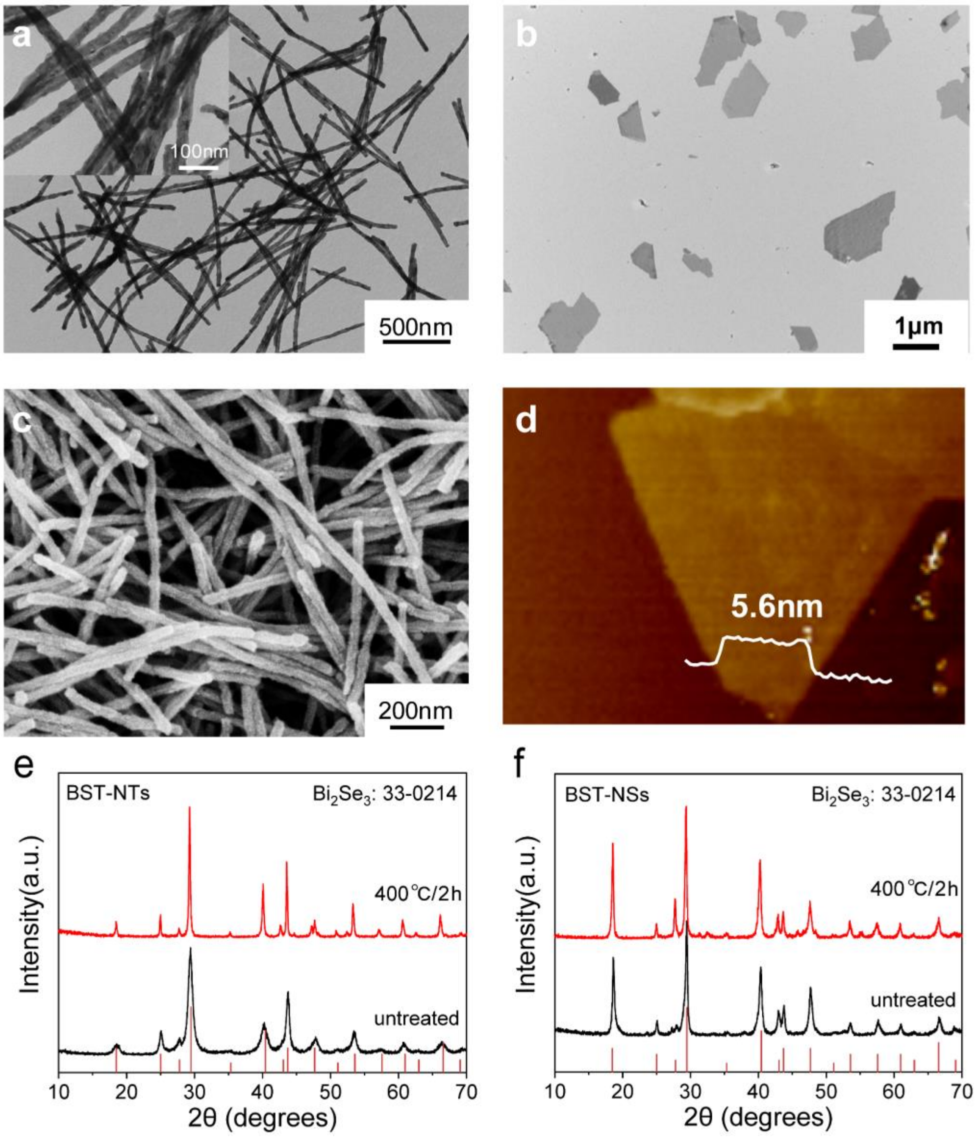


**Supplementary Figure 8 Characterization of BST-NTs and BST-NSs. a** and **b**, TEM images of BST-NTs and BST-NSs. **c**, SEM image of BST-NTs. **d**, AFM image of BST-NSs. **e** and **f**, XRD patterns of BST-NTs and BST-NSs before and after annealing at 400 ℃ for 2 h.

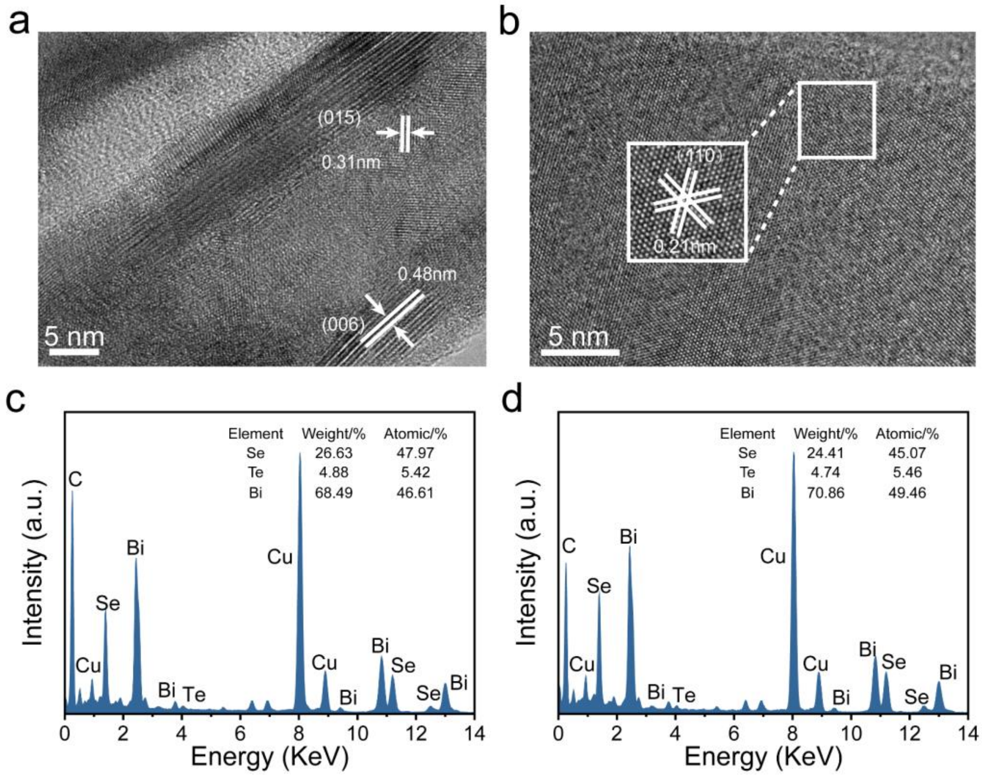


**Supplementary Figure 9 HRTEM and EDS analyses of BST-NTs and BST-NSs. a**, **b**, HRTEM images of BST-NTs and BST-NSs. **c**, **d**, EDS spectra of BST-NTs and BST-NSs.

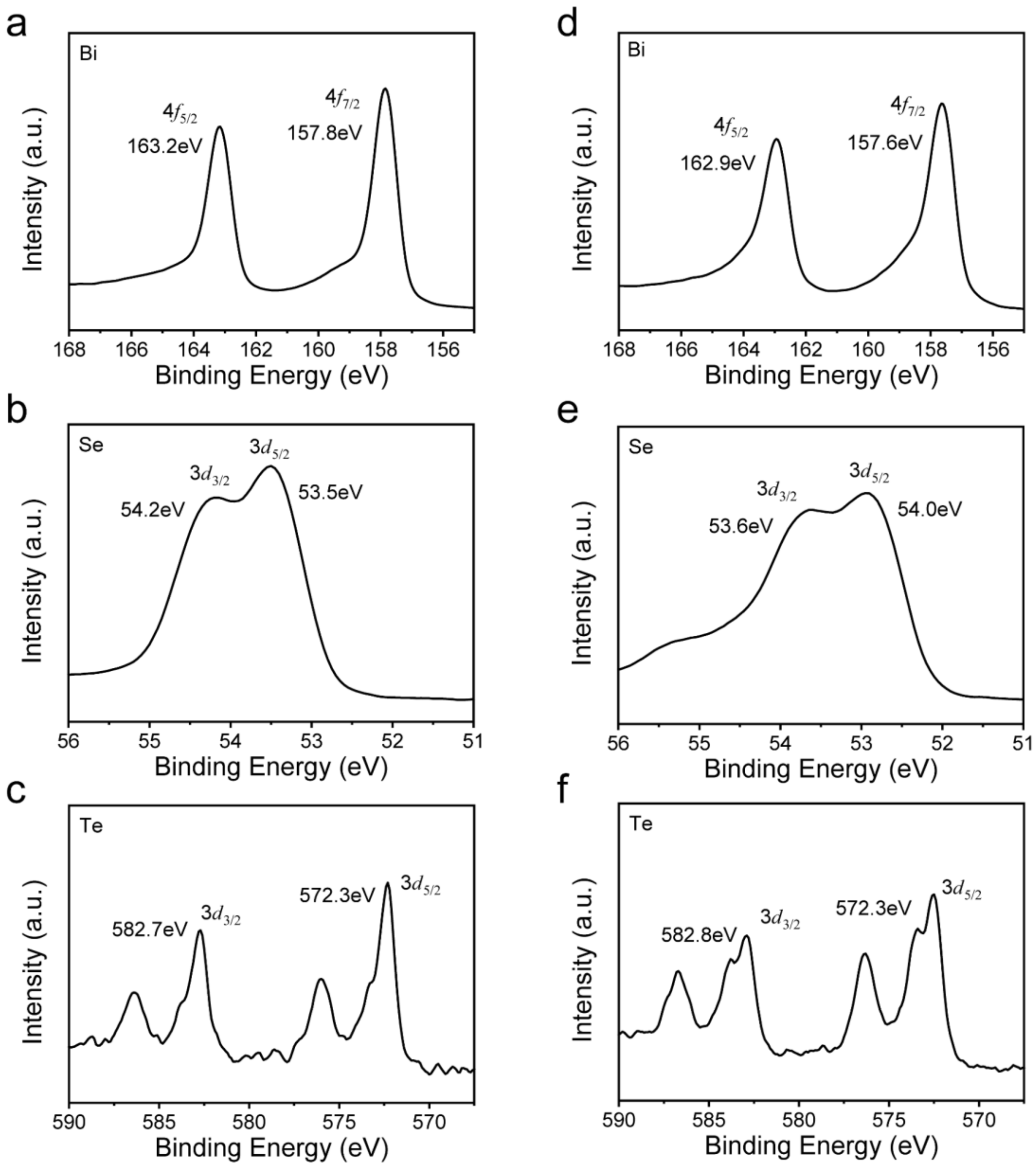


**Supplementary Figure 10 XPS spectra of BST-NTs (a-c) and BST-NSs (d-f).**

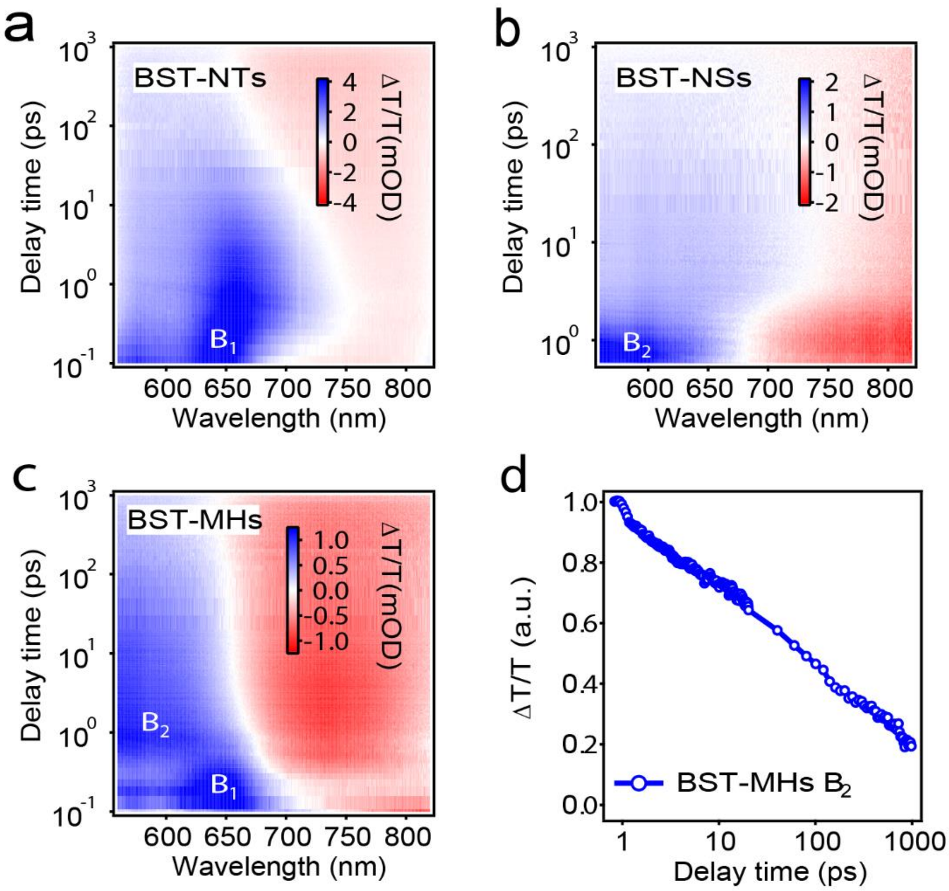


**Supplementary Figure 11 Spectroscopic characterization of photoanodes. a-c**, Color plot of TA spectra of BST-NTs (**a**), BST-NSs (**b**), and BST-MHs (**c**). The positive peaks $B_1$ and $B_2$ correspond to particular state filling of BST-NTs and BST-NSs, respectively. **d**, Kinetics of $B_2$ in BST-MHs.

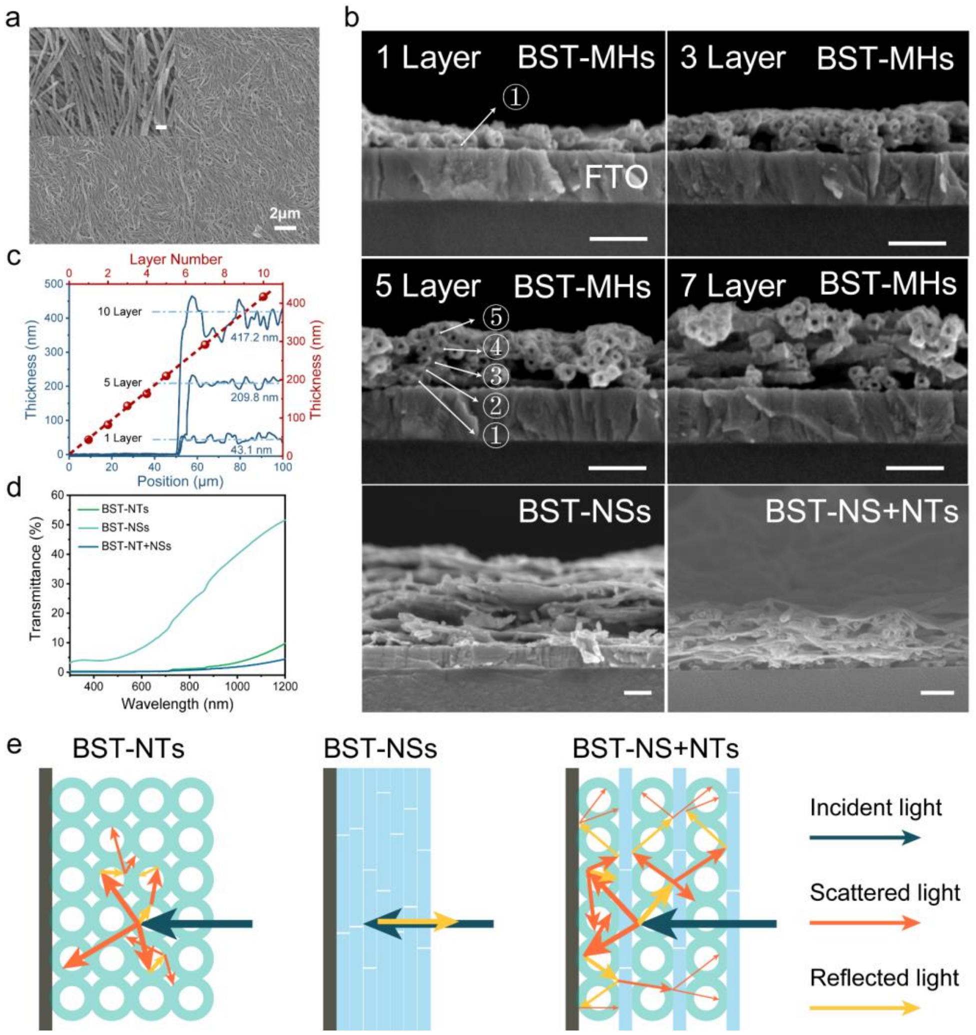


**Supplementary Figure 12 Characterizations of photoanodes. a**, SEM image of the BST-MH photoanode. **b**, SEM images of the cross-section of the BST-MH, BST-NS and BST-NT photoanodes. The scale bar is 200 nm. **c**, The thicknesses of the BST-MH photoanodes with varying numbers of layers. **d,** Transmittance spectrum of BST-NT, BST-NS and BST-NT + NS photoanodes. **e,** Schematic illustration of reflection and scattering effects in BST-NT, BST-NS and BST-NT+NS photoanodes.

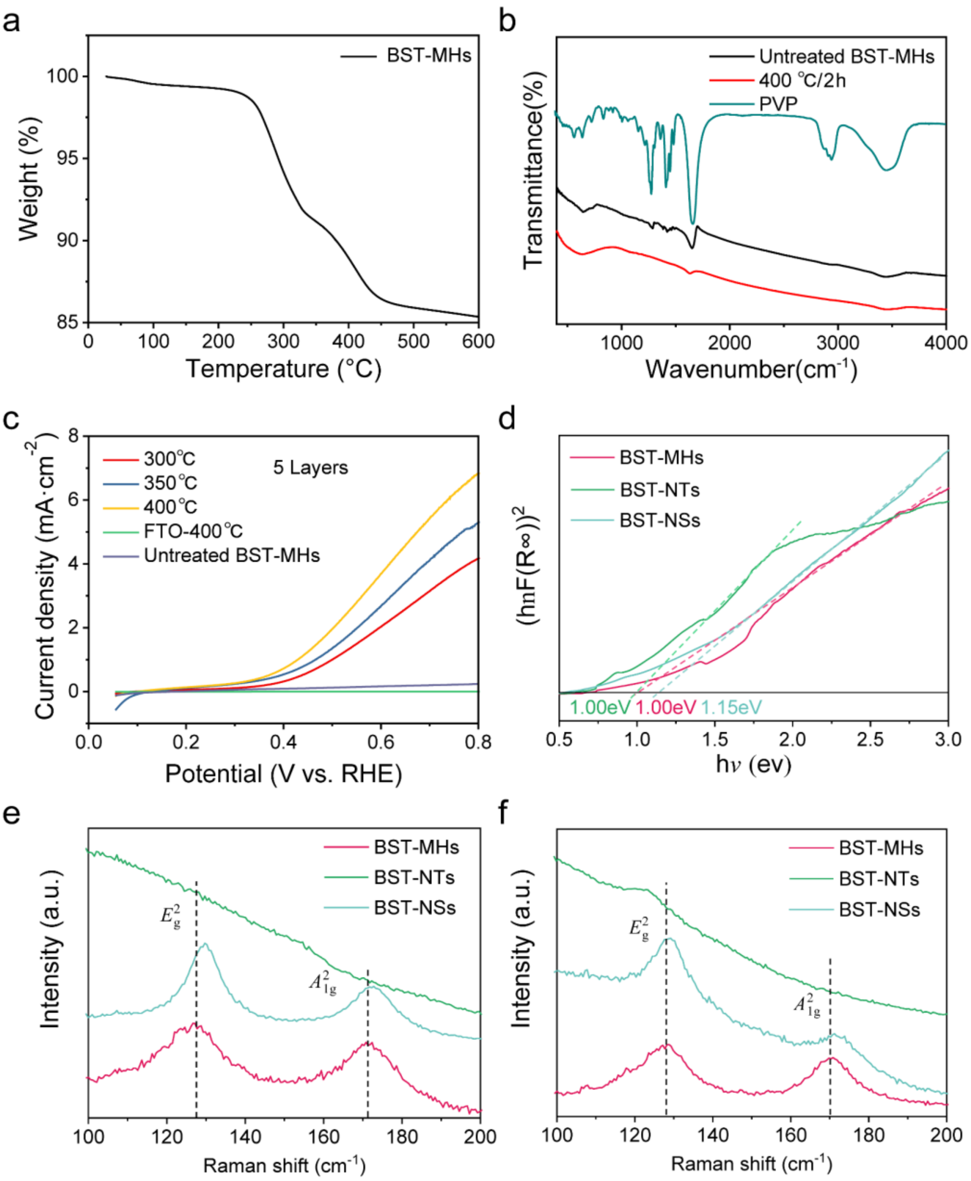


**Supplementary Figure 13 Characterizations and PEC measurements of annealed BST samples. a**, Thermal gravimetric analysis (TGA) of BST-MHs, the results reveal that PVP pyrolyzes at 300 ℃. **b**, The Fourier transform infrared (FT-IR) spectra of BST-MHs before and after annealing at 400 ℃ for 2 h. The results show that the infrared characteristic peaks of PVP in annealed samples become smaller than the untreated samples. **c**, Current-potential curves of BST-MH photoanodes annealed at different temperature for 2 h and FTO glass annealed at 400 °C for 2 h. The current-potential curves were measured in an electrolyte solution containing 0.08 mol $L^{-1}$ $Na_2S$ and 0.50 mol $L^{-1}$ $Na_2SO_3$ as hole scavengers (pH = 12.9) under visible light irradiation ($\lambda$ > 420 nm, 100 mW $cm^{-2}$). **d**, Tauc plots of BST-MHs, BST-NTs, and BST-NSs after

annealing at 400 ℃ for 2 h. **e**, **f**, Raman spectra of BST-MHs, BST-NTs, and BST-NSs without any post-treatment (**e**) and after annealing at 400 ℃ for 2 h (**f**).

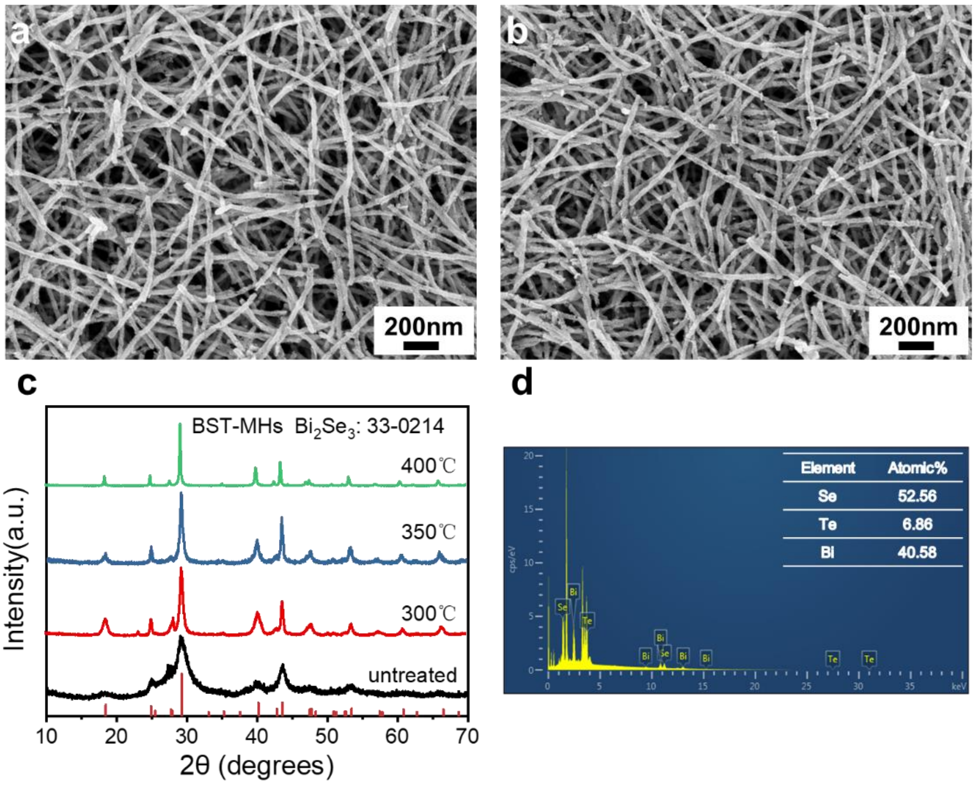


**Supplementary Figure 14 SEM images and XRD patterns of annealed BST-MHs.** **a**, SEM image of the BST-MHs without any post-treatment. **b**, SEM image of the BST-MHs after annealing at 400 ℃ for 2 h. **c**, XRD patterns of BST-MHs after annealing at different temperatures for 2 h. **d**, EDS spectrum of BST-MHs after annealing at 400 ℃ for 2 h.

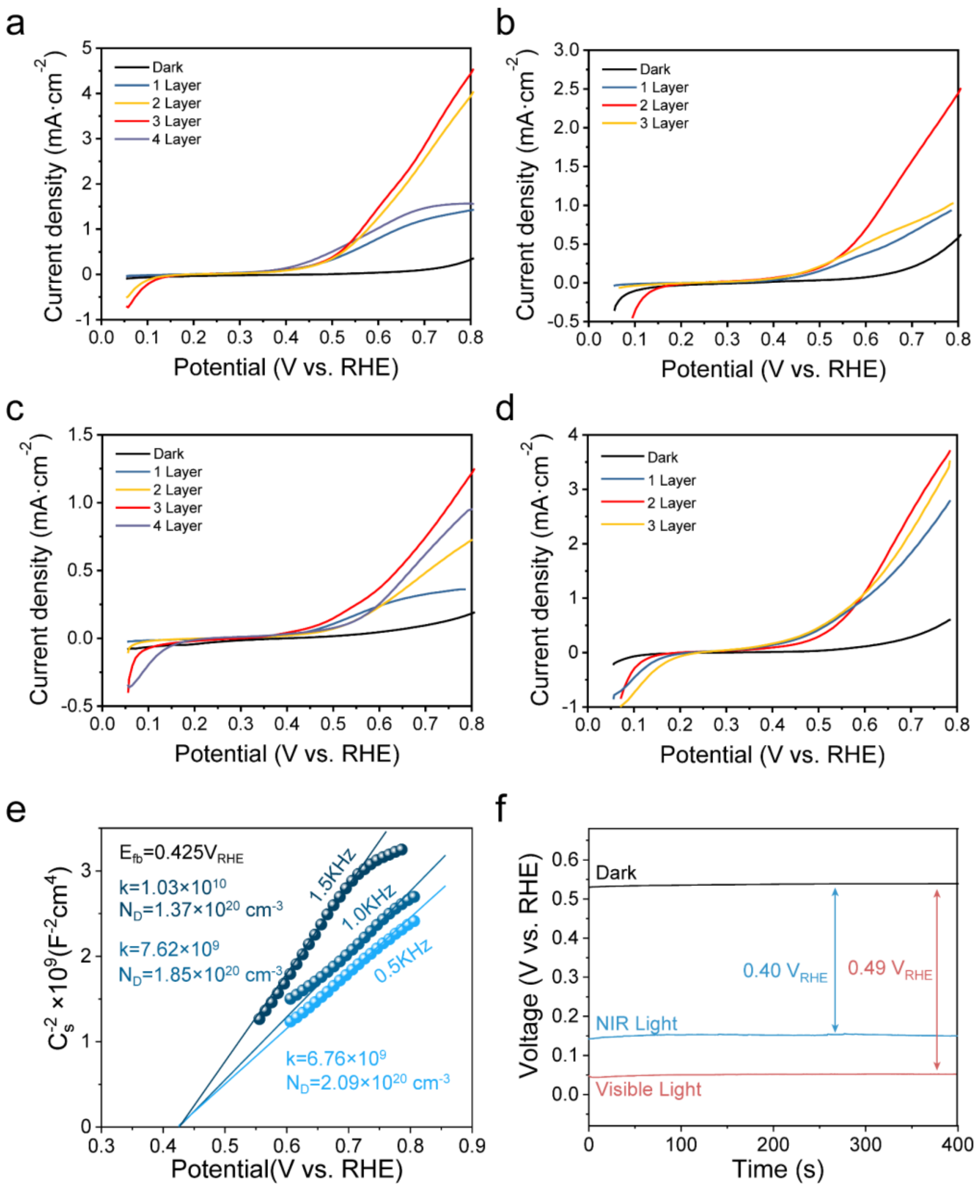


**Supplementary Figure 15 PEC performances of photoanode. a-d**, Current-potential curves of BST-MH (**a**), BST-NT (**b**), BST-NS (**c**), and BST-NT+NS (**d**) photoanodes with different layers under NIR light irradiation ($\lambda$ > 800 nm, 100 mW cm$^{-2}$). **e**, Mott−Schottky plots of BST-MH photoanodes collected at different frequencies. **f,** Open circuit potential measurements under different solar source ($\lambda$ > 420 nm, $\lambda$ > 800 nm). All the experiments were measured in an electrolyte solution containing 0.08 mol L$^{-1}$ $Na_2S$ and 0.50 mol L$^{-1}$ $Na_2SO_3$ as hole scavengers (pH = 12.9).

Given the basic device physics, photoanode thickness is limited to the carrier collection length (including both the diffusion length and the width of the electric-field

region) and the absorption depth of the material for the relevant photon energies of the solar spectrum.[1] Hence, we investigate the influence of the photoanode thickness on the PEC performances, as shown in Supplementary Figure 15. As the thickness of BST-MHs increases, the corresponding photocurrent density is slightly enhanced. As a result, the optimal thicknesses for BST-MHs, NTs, NSs, and NTs+NSs are determined to be 3, 2, 3 and 2 layers, respectively. When electrode thickness is further increased beyond the optimum value, the photocurrent density decreases.

Upon illumination, the semiconductor will generate light-induced charges, and the Fermi level of the semiconductor will split into separate quasi-Fermi levels of electrons and holes. The quasi-Fermi level splitting produces an open-circuit photovoltage that is equal to the difference between the two quasi-Fermi levels. Therefore, the magnitude of the photovoltage is directly related to the extent of separation between quasi-Fermi levels.

In our measurements of photovoltage, the illumination intensity of visible and NIR light is 100 mW $cm^{-2}$. However, from the analysis of UV-vis-IR absorption spectrum, it can be concluded that BST-MHs have a better absorption capacity for visible light. Therefore, although NIR light has the same illumination intensity as visible light, the BST-MH photoanode absorbs more photons from the visible region to generate light-induced charges, resulting in a larger difference between the quasi-Fermi levels.

Based on the above analysis, the photovoltage of BST-MHs should be different under the irradiation of different light sources, which is mainly affected by the difference in the absorption capacity of BST-MH to light of different wavelengths (Supplementary Fig. 15f).

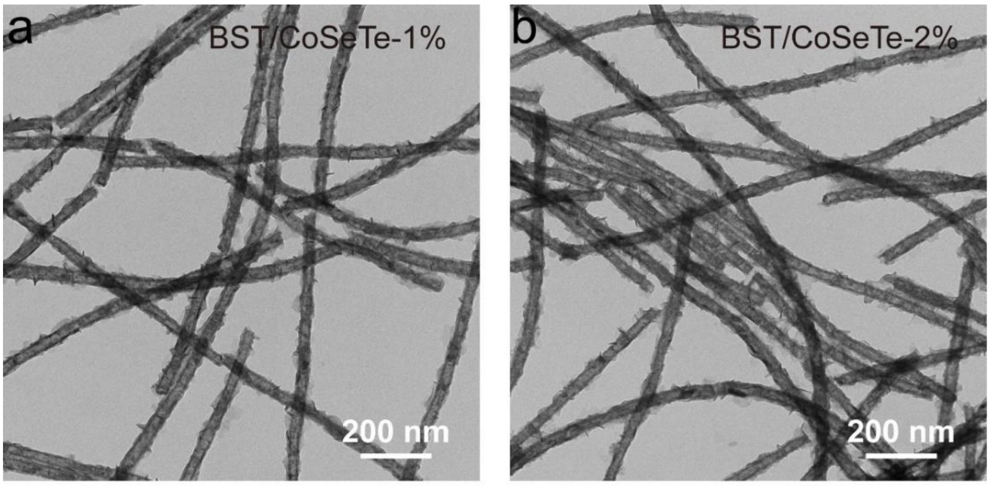


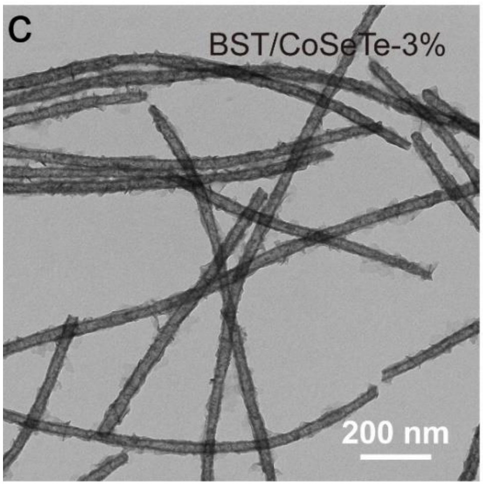


d

| Sample | Co/Bi ratio (%) |
|---|---|
| BST/CoSeTe-1% | 1.18 |
| BST/CoSeTe-2% | 1.99 |
| BST/CoSeTe-3% | 3.32 |

**Supplementary Figure 16 Characterization of BST/CoSeTe. a-c,** TEM images of BST/CoSeTe-1% (**a**), BST/CoSeTe-2% (**b**), BST/CoSeTe-3% (**c**). **d,** Co/Bi ratio of BST/CoSeTe-1%, BST/CoSeTe-2%, BST/CoSeTe-3%.

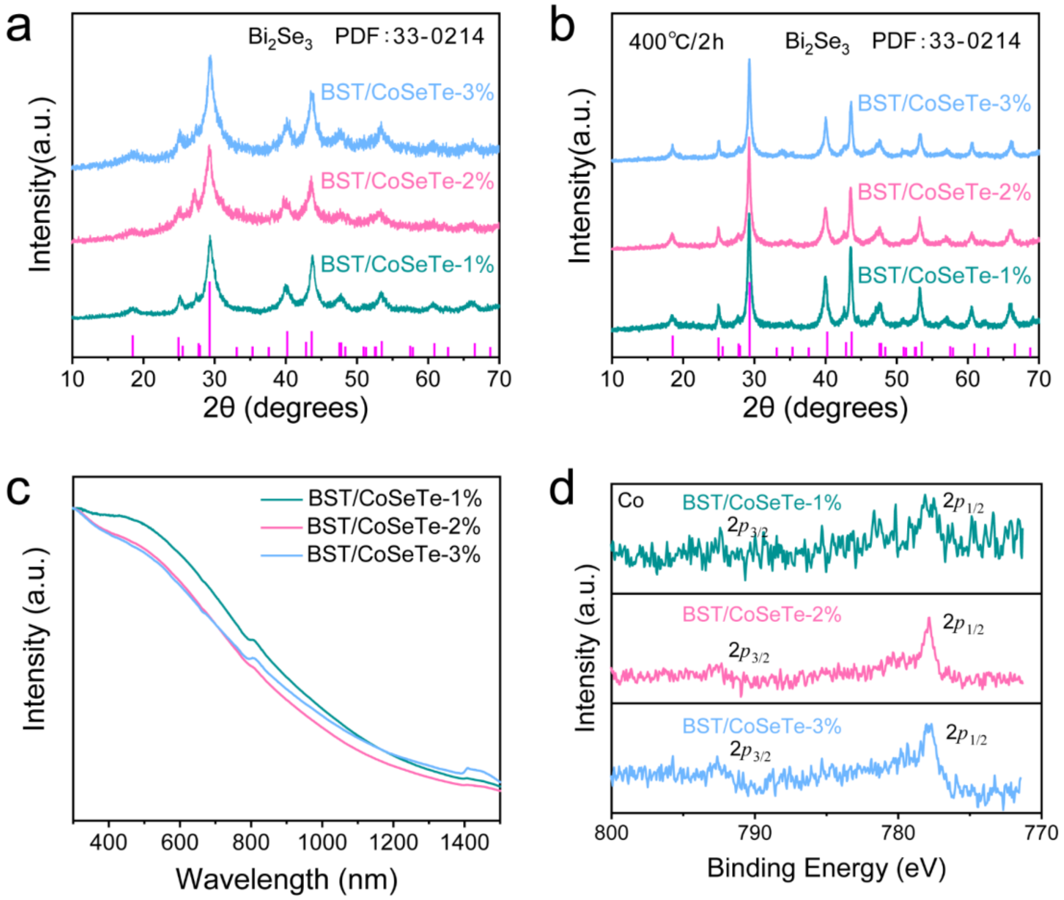


**Supplementary Figure 17 Characterization of BST/CoSeTe. a, b,** XRD patterns of BST/CoSeTe-1%, BST/CoSeTe-2%, BST/CoSeTe-3% before (**a**) and after annealing at 400 ℃ for 2 h (**b**). **c, d,** UV-Vis-NIR spectra (**c**) and XPS spectra (**d**) of BST/CoSeTe-1%, BST/CoSeTe-2%, BST/CoSeTe-3%.

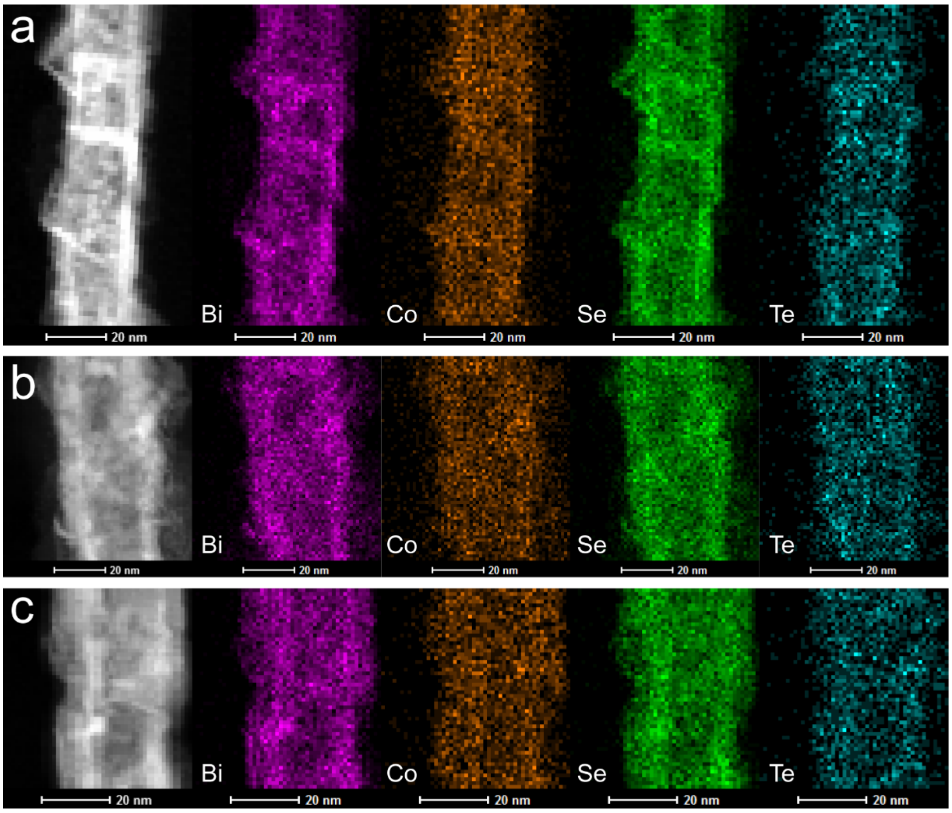


**Supplementary Figure 18 Characterization of BST/CoSeTe. a-c,** EDS mapping images of BST/CoSeTe-1% (**a**), BST/CoSeTe-2% (**b**), BST/CoSeTe-3% (**c**).

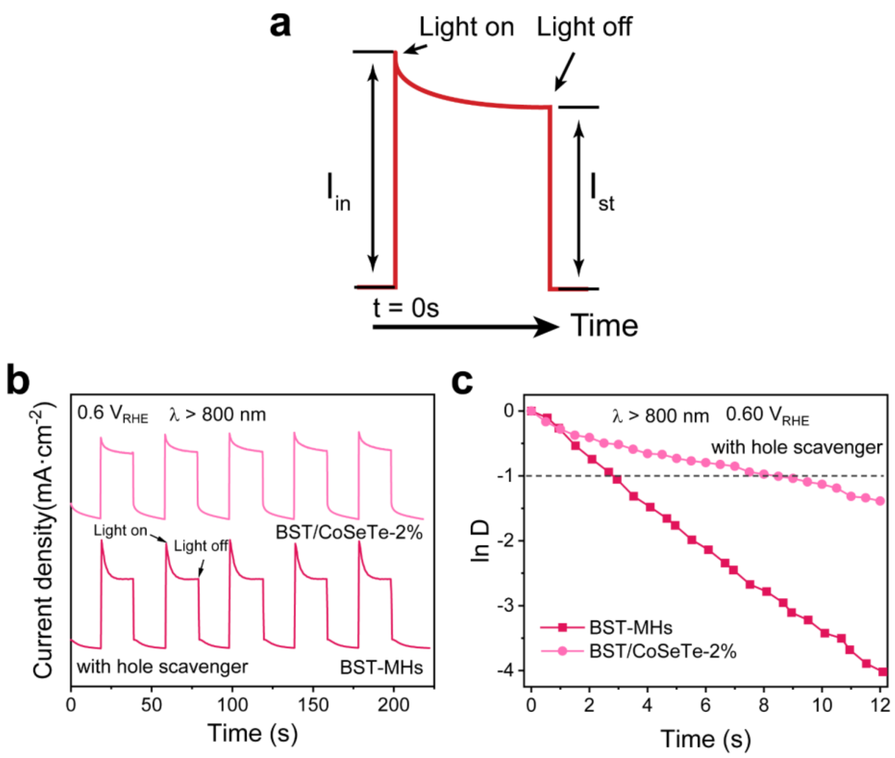


**Supplementary Figure 19 Transient photocurrent analysis. a,** Schematic of a typical photocurrent transient response. **b,** Transient current-time curves of BST-MH and BST/CoSeTe-2% photoanodes at 0.60 $V_{RHE}$ under NIR light irradiation ($\lambda$ > 800 nm, 100 mW cm$^{-2}$). **c,** Normalized plots of photocurrent density-time dependence of the BST-MH and BST/CoSeTe-2% photoanodes at 0.6 $V_{RHE}$. The transient photocurrent was measured in an electrolyte solution containing 0.08 mol L$^{-1}$ $Na_2S$ and 0.50 mol L$^{-1}$ $Na_2SO_3$ as hole scavengers (pH = 12.9).

The spike in transient current is related to the slow surface reaction kinetics of photogenerated carriers. As shown in Supplementary Fig. 19a, upon optical excitation, the semiconductor generates electron-hole pairs and gives rise to a large photocurrent spike ($I_{in}$) instantaneously. The photogenerated carriers usually suffer from severe recombination when the efficiencies of charge separation, transfer, and surface reactions are low. As a result, the transient photocurrent decays with time. Only when the charge-generation and recombination rates reach equilibrium, a steady-state photocurrent ($I_{st}$) can be achieved. Therefore, under the same conditions, the current decay rate translates into the carrier recombination rate in the photoelectrode.

To quantitatively compare the carrier lifetime in the photoelectrodes, we study the transient decay time $\tau$ through the logarithmic plot of the parameter $D$. The value of $D$ is calculated as:

$$D = (I_t - I_{\text{st}})/(I_{\text{in}} - I_{\text{st}}) \quad (1)$$

where $I_t$ represents the photocurrent at time $t$(s), and $I_{\text{st}}$ is the steady-state current. We compare the transient decay time $\tau$ at ln$D$ = -1.

As shown in Fig. 3e, the transient decay time $\tau$ of the BST-MH photoanode is the largest compared with those of BST-NT, BST-NS, and BST-NT+NS photoanodes, indicating that the lattice-matched morphological heterojunctions can suppress carrier recombination.

To further suppress carrier recombination, we sought to accelerate the hole-involved oxidation reaction kinetics by fabricating the BST/CoSeTe photoanode (Supplementary Fig. 16-18), wherein CoSeTe serves as the oxidation cocatalyst. The transient current spike of the BST/CoSeTe photoanode is significantly smaller than that of the BST-MH photoanode (Supplementary Fig. 19b). The normalized transient decay curves further verify that the spike in BST-MHs originates from inefficient anodic oxidation reaction, and introducing CoSeTe can help to mitigate this problem, extending the transient decay time τ three-fold (Supplementary Fig. 19c).

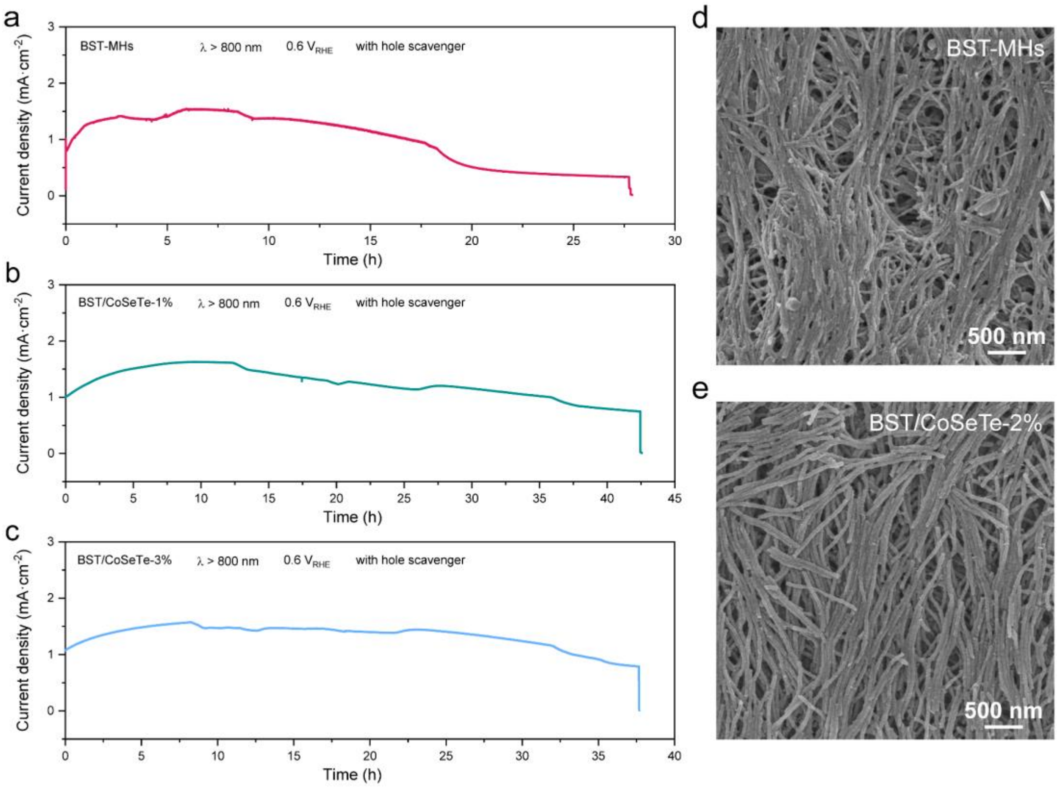


**Supplementary Figure 20 PEC stability. a-c,** Stability test of the BST-MH (**a**), BST/CoSeTe-1% (**b**), and BST/CoSeTe-3% (**c**) photoanodes at 0.60 $V_{RHE}$ under NIR light irradiation ($\lambda$ > 800 nm, 100 mW $cm^{-2}$). **d, e,** SEM images of the BST-MHs (**d**) and BST/CoSeTe-2% (**e**) after PEC $H_2$ evolution. All the experiments were measured in an electrolyte solution containing 0.08 mol $L^{-1}$ $Na_2S$ and 0.50 mol $L^{-1}$ $Na_2SO_3$ as hole scavengers (pH = 12.9).

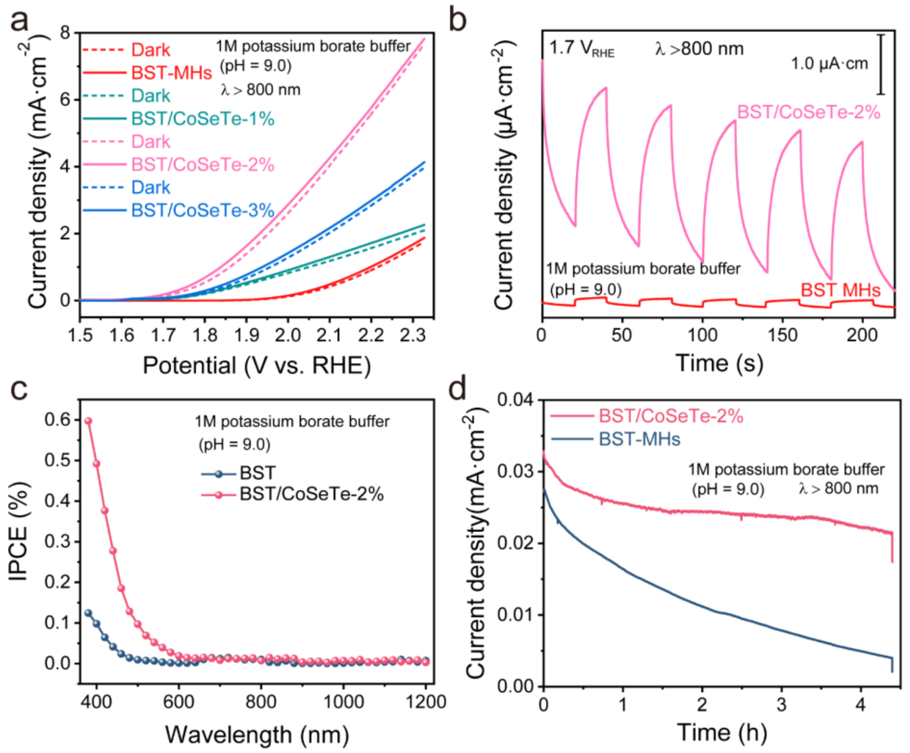


**Supplementary Figure 21 PEC water splitting. a,** Current-potential curves of BST-MHs, BST/CoSeTe-1%, BST/CoSeTe-2%, and BST/CoSeTe-3% photoanodes under NIR light irradiation ($\lambda$ > 800 nm, 100 mW cm$^{-2}$). **b-d,** Transient current-time curves (b), IPCEs (c), and Stability (d) of the BST-MH and BST/CoSeTe-2% photoanodes at 1.70 $V_{RHE}$ under NIR light irradiation ($\lambda$ > 800 nm, 100 mW cm$^{-2}$). All experiments were measured in an electrolyte solution containing 1M potassium borate buffer (pH = 9).

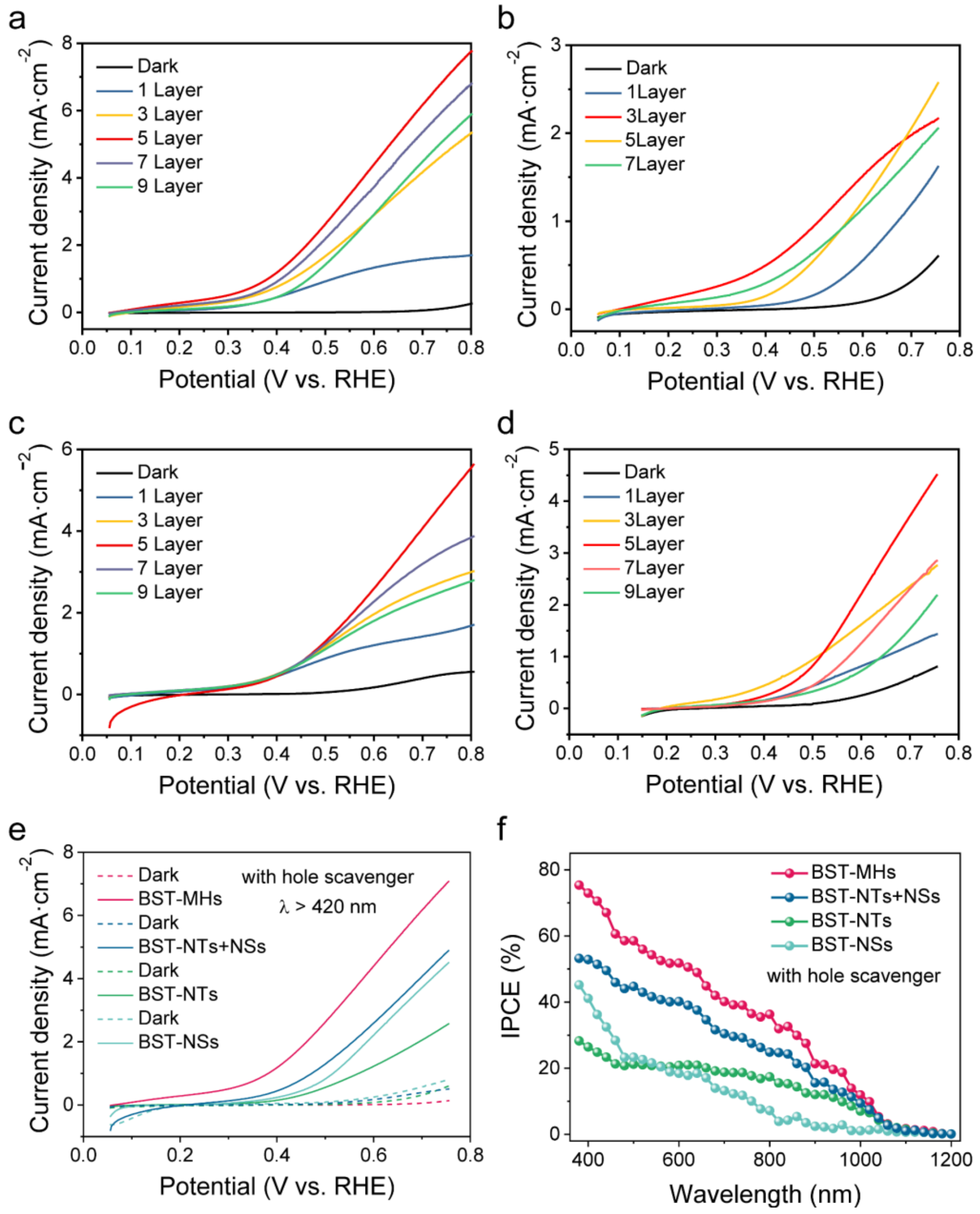


**Supplementary Figure 22 PEC performances of photoanodes. a-d**, Current-potential curves of BST-MH (**a**), BST-NT (**b**), BST-NS (**c**), and BST-NT+NS (**d**) photoanodes with different layers under visible light irradiation ($\lambda$ > 420 nm, 100 mW cm$^{-2}$). **e, f**, Current-potential curves (**e**) and IPCEs (**f**) of the BST-MH, BST-NT, BST-NS, and BST-NT+NS photoanodes under visible light irradiation ($\lambda$ > 420 nm, 100 mW cm$^{-2}$). All the experiments were measured in an electrolyte solution containing 0.08 mol L$^{-1}$ $Na_2S$ and 0.50 mol L$^{-1}$ $Na_2SO_3$ as hole scavengers (pH = 12.9).

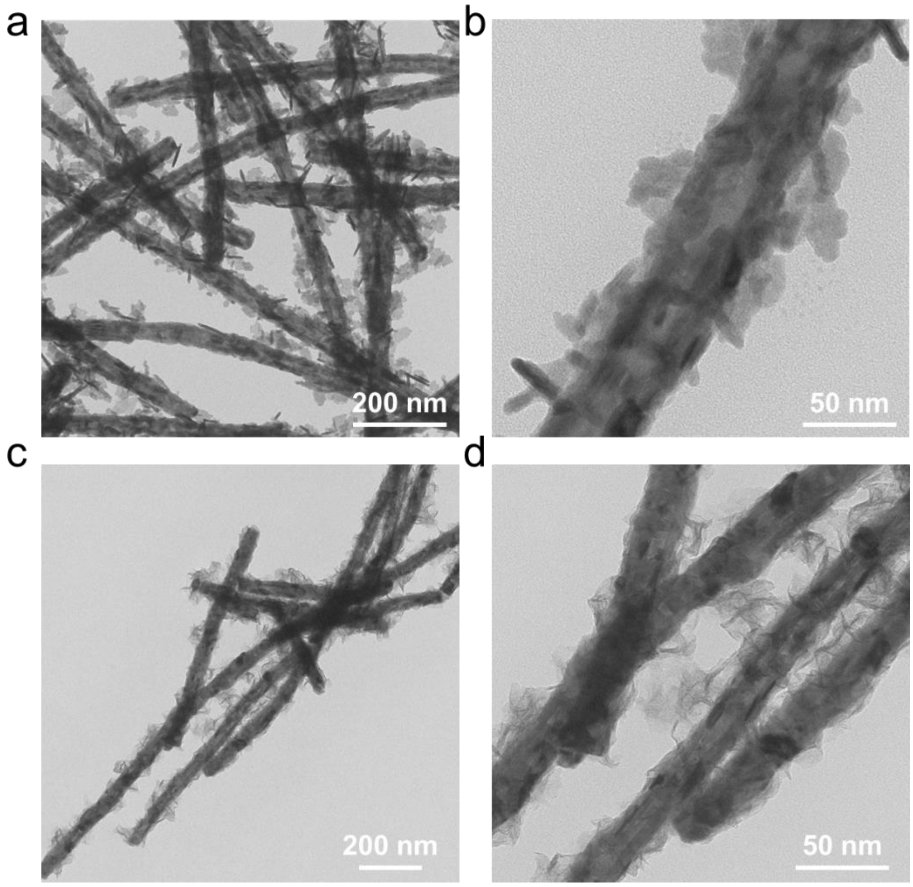


**Supplementary Figure 23 Characterization of the BST/$Bi_2S_3$ and BST/$MoS_2$. a**, **b**, TEM images of BST/$Bi_2S_3$. **c**, **d**, TEM images of BST/$MoS_2$.

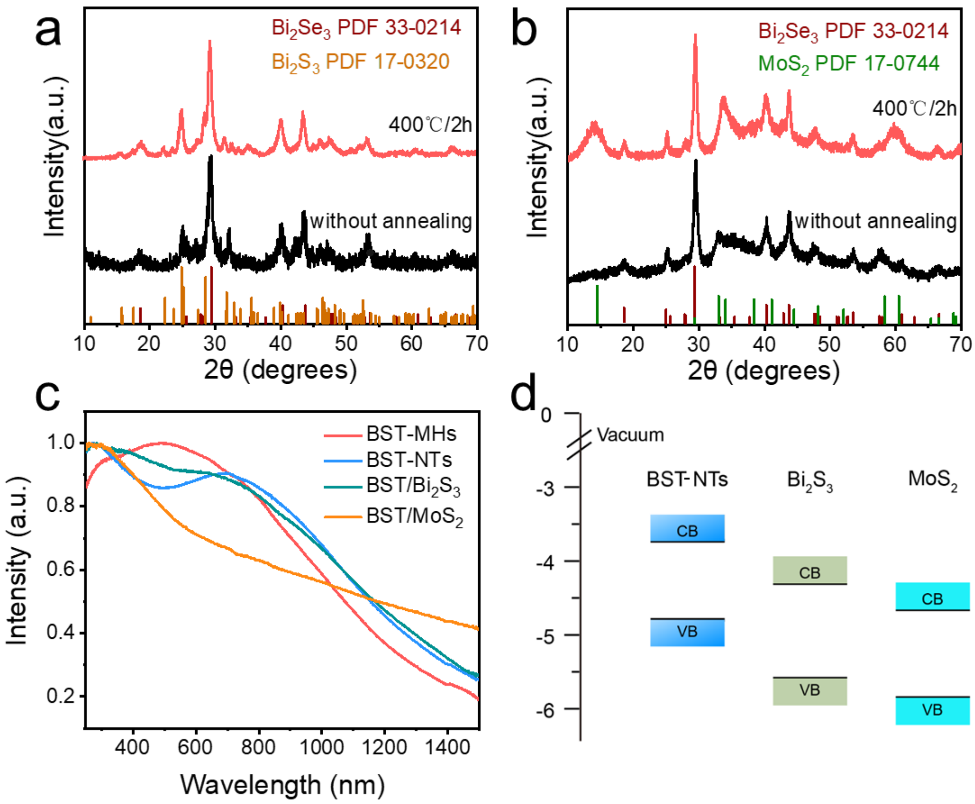


**Supplementary Figure 24 Characterization of the BST/$Bi_2S_3$ and BST/$MoS_2$. a**, **b**, XRD patterns of BST/$Bi_2S_3$ and BST/$MoS_2$ before and after annealing at 400 ℃ for 2 h. **c**, UV-Vis-NIR spectra of BST-MHs, BST-NTs, BST/$Bi_2S_3$, and BST/$MoS_2$. **d**, Energy band diagrams of BST-MHs, BST/$Bi_2S_3$, and BST/$MoS_2$.

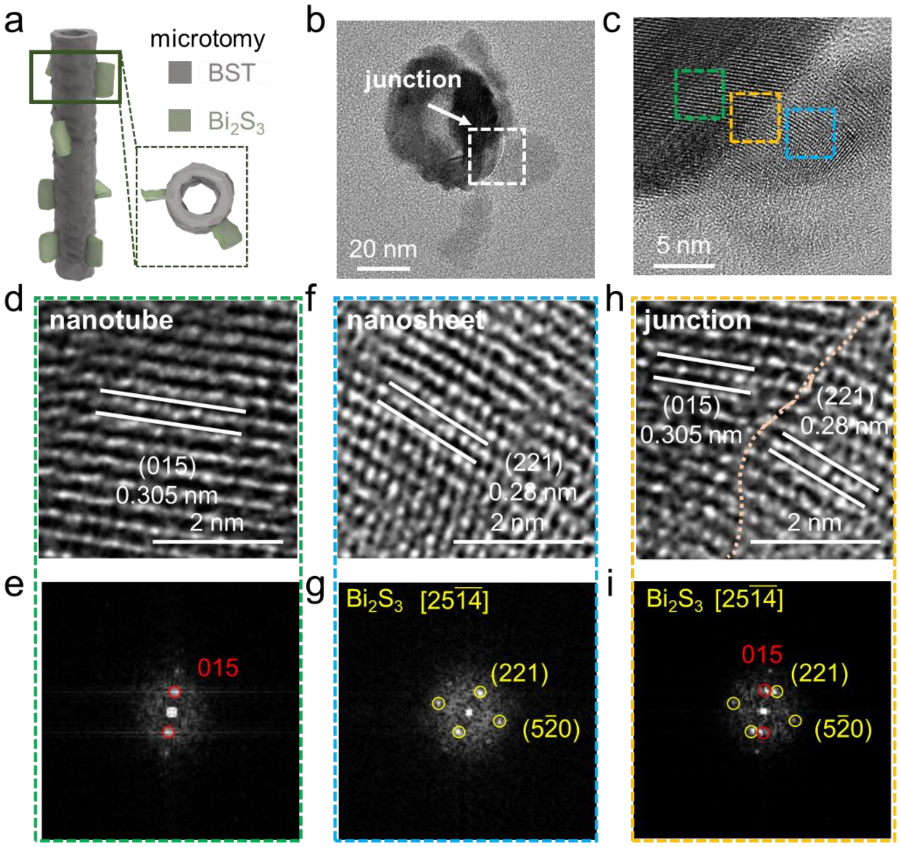


**Supplementary Figure 25 Characterization of the junction between BiSeTe nanotubes and $Bi_2S_3$ nanosheets.** **a**, Schematic diagram of the sliced sample. **b**, **c**, TEM and HRTEM images of the junction. **d**, **e**, HRTEM image and FFT of BiSeTe nanotube in the green domain of (**c**). **f**, **g**, HRTEM image and FFT of $Bi_2S_3$ nanosheet in the blue domain of (**c**). **h**, **i**, HRTEM image and FFT of junction in the yellow domain of (**c**).

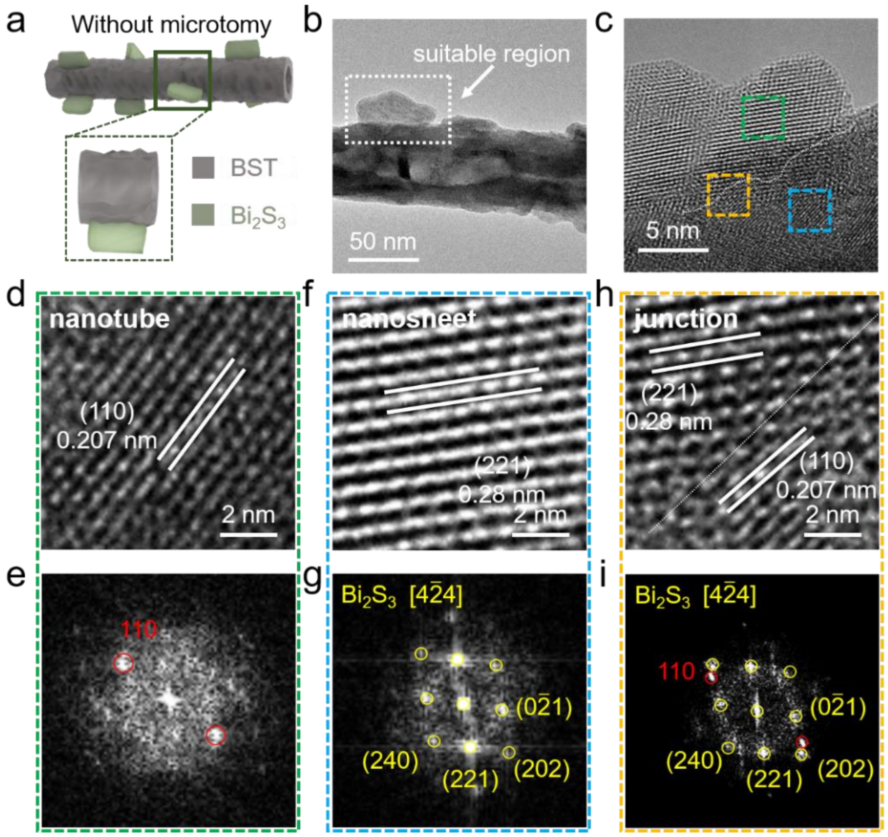


**Supplementary Figure 26 Characterization of the junction between BiSeTe nanotubes and $Bi_2S_3$ nanosheets. a**, Schematic diagram of the junction. **b**, **c**, TEM and HRTEM images of the junction. **d**, **e**, HRTEM image and FFTs of BiSeTe nanotube in the green domain of (**c**). **f**, **g**, HRTEM image and FFT of $Bi_2S_3$ nanosheet in the blue domain of (**c**). **h**, **i**, HRTEM image and FFT of junction in the yellow domain of (**c**).

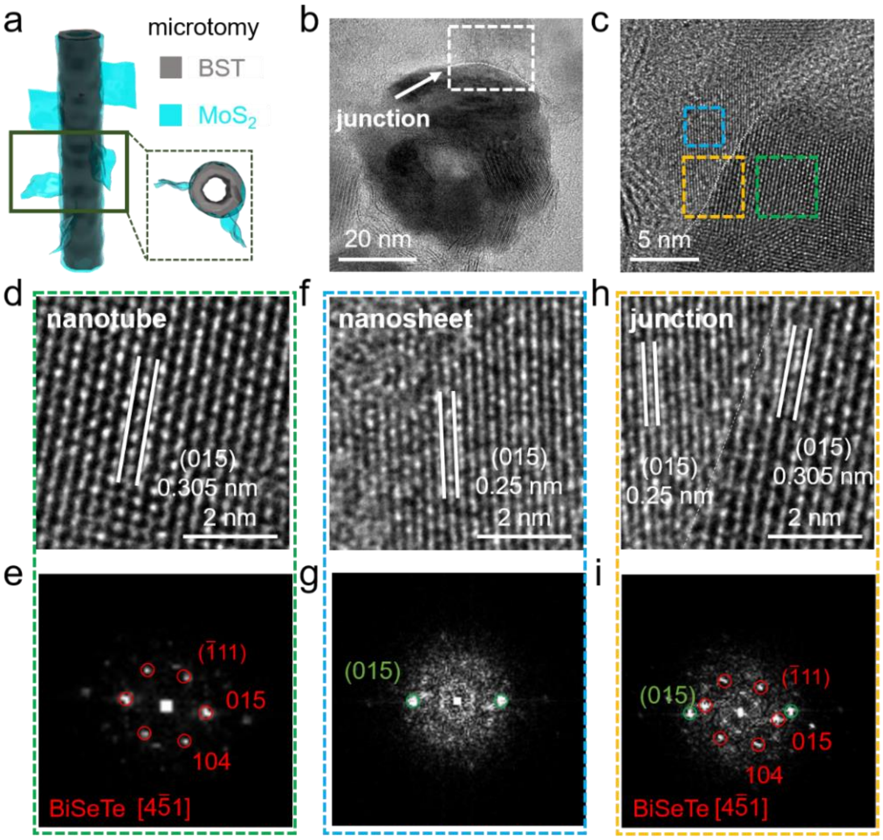


**Supplementary Figure 27 Characterization of the junction between BiSeTe nanotubes and $MoS_2$ nanosheets.** **a**, Schematic diagram of the sliced sample. **b**, **c**, TEM and HRTEM images of the junction. **d**, **e**, HRTEM image and FFT of BiSeTe nanotube in the green domain of (**c**). **f**, **g**, HRTEM image and FFT of $MoS_2$ nanosheet in the blue domain of (**c**). **h**, **i**, HRTEM image and FFT of junction in the yellow domain of (**c**).

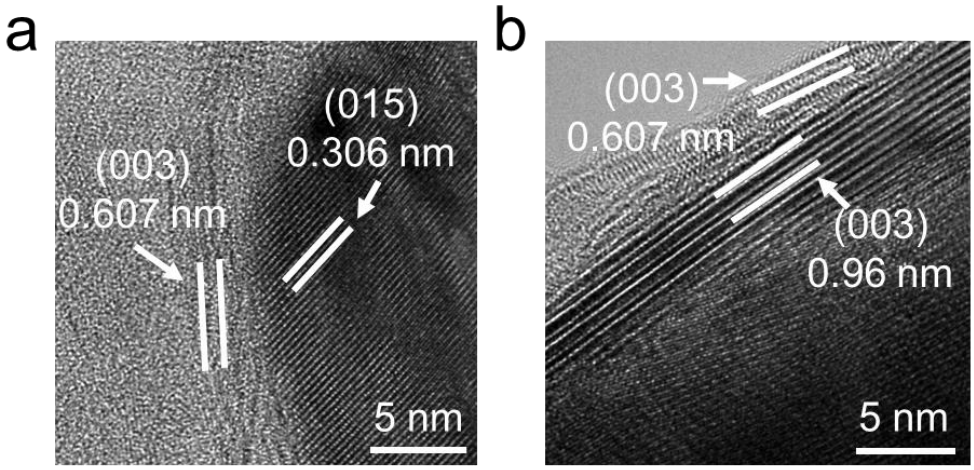


**Supplementary Figure 28 Characterization of the junction between BiSeTe nanotubes and $MoS_2$ nanosheets.** HRTEM images of the junction between BiSeTe nanotubes and $MoS_2$ nanosheets.

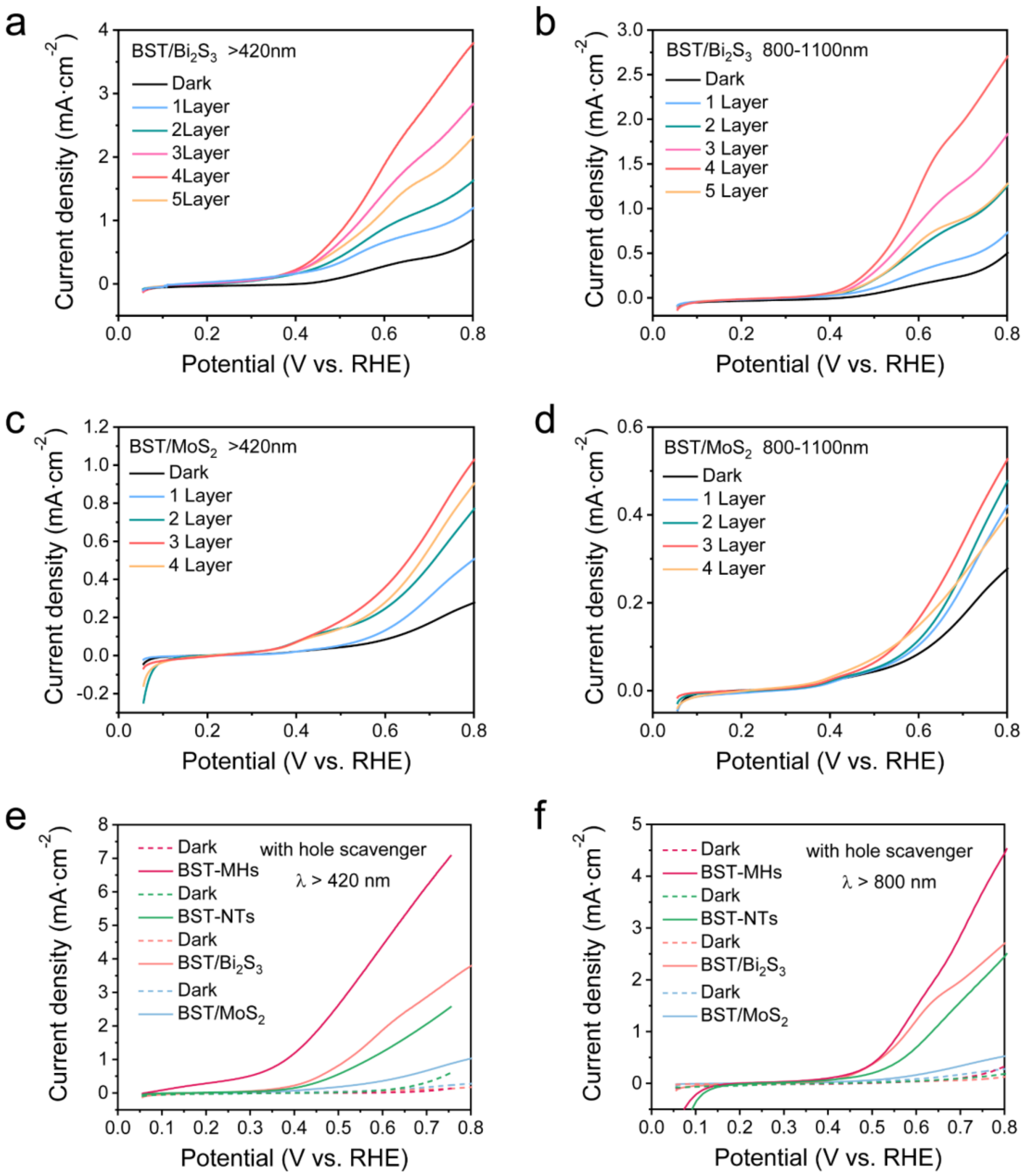


**Supplementary Figure 29 PEC performances of BST/$Bi_2S_3$ and BST/$MoS_2$ photoanodes. a-d**, Current-potential curves of BST/$Bi_2S_3$ (**a** and **b**), BST/$MoS_2$ (**c** and **d**) photoanodes with different layers under visible light irradiation ($\lambda$ > 420 nm, 100 mW cm$^{-2}$) and NIR light irradiation ($\lambda$ > 800 nm, 100 mW cm$^{-2}$). **e, f**, Current-potential curves of BST-MHs, BST-NTs, BST/$Bi_2S_3$, and BST/$MoS_2$ photoanodes under visible light irradiation (**e**, $\lambda$ > 420 nm, 100 mW cm$^{-2}$) and NIR light irradiation (**f**, $\lambda$ > 800 nm, 100 mW cm$^{-2}$). All the experiments were measured in an electrolyte solution containing 0.08 mol L$^{-1}$ $Na_2S$ and 0.50 mol L$^{-1}$ $Na_2SO_3$ as hole scavengers (pH = 12.9).

In order to better prove the function of a lattice-matched heterojunction, we further grew another kind of Bi-based chalcogenides ($Bi_2S_3$ nanosheets) and 2D semiconductor ($MoS_2$ nanosheets) on the BiSeTe nanotubes (Supplementary Fig. 23-24). Large differences in lattice spacing and crystalline orientation can be found from HRTEM images at interfaces (Supplementary Fig. 25-28). Specifically, FFTs of the interfacial regions show two diffraction patterns that can be assigned to nanotubes and nanosheets, indicating the existence of lattice mismatch at interfaces. Then, we examined their PEC performances under the same conditions as the BST-MHs (Supplementary Fig. 29). Under both NIR irradiation and visible light irradiation, the photo-current densities of BST/$Bi_2S_3$ are slightly higher than that of plain nanotubes, but much lower than that of lattice-matched BST-MHs. We attribute this result to the somewhat impeded charge separation due to interfacial defects, which act as recombination centers, and therefore deteriorated device performances. The influence of interfacial defects in lattice-mismatched heterojunctions is more obvious in the case of BST/$MoS_2$ heterostructures. The abundance of interfacial defects in BST/$MoS_2$ leads to negligible PEC performances. All above experimental results provide evidence that lattice-matched hetero-interfaces benefit charge separation by reducing interfacial defects.

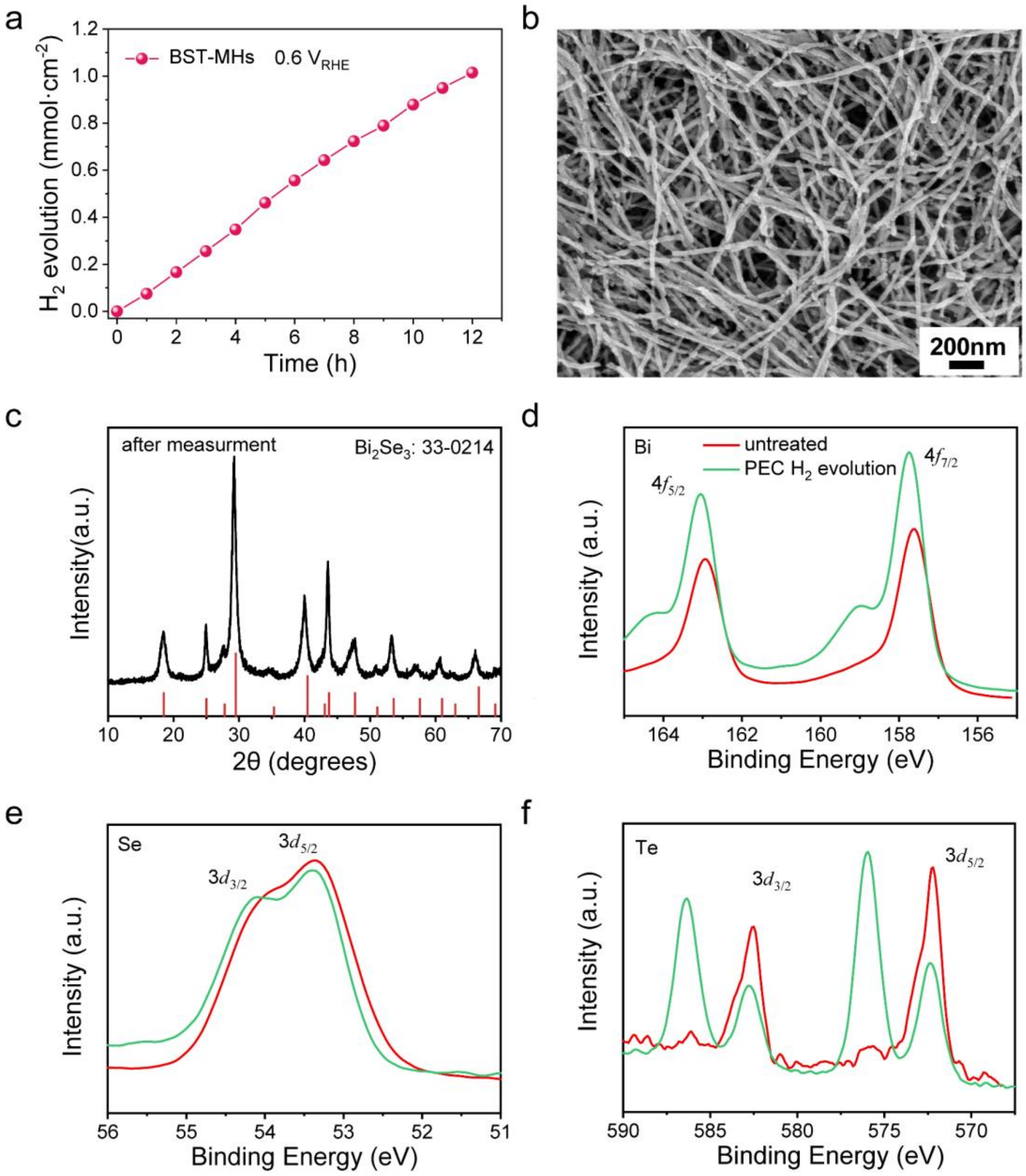


**Supplementary Figure 30 PEC $H_2$ evolution and characterization of BST-MHs post-experiment. a**, $H_2$ evolution and photocurrent density of the BST-MH photoanode at 0.60 $V_{RHE}$. All the experiments were measured in an electrolyte solution containing 0.08 mol $L^{-1}$ $Na_2S$ and 0.50 mol $L^{-1}$ $Na_2SO_3$ as hole scavengers (pH = 12.9) under visible light irradiation ($\lambda$ > 420 nm,100 mW $cm^{-2}$). **b**, **c**, SEM image (**b**) and XRD pattern (**c**) of the BST-MHs after PEC $H_2$ evolution. **d-f**, XPS spectra of BST-MHs pre- and post-experiment.

**Supplementary Table 1 Summary of the PEC performances of different NIR photoanodes.**

| Catalyst | Spectral range (nm) | $J_{NIR}$ (mA cm$^{-2}$) | IPCE | Causes for low efficiency |
|---|---|---|---|---|
| Up-converting phosphor semiconductors | >700 nm | <0.1 | NA | Low efficiency of up-conversion luminescence |
| Metal plasmonic nanostructures/ semiconductors | >700 nm | <0.1 | < 1% | Short lifetime of hot carriers |
| Narrow bandgap semiconductors | >700 nm | <2 | 12% | Short lifetime of photoexcited carriers |
| **BST-MHs** | **800-1100 nm** | **1.5** | **36%** | |

**Supplementary Table 2 Fitting lifetimes of TA kinetics.**

| Kinetics | Fitting Range 1 | Fitting Function 1 | Lifetime 1 | Fitting Range 2 | Fitting Function 2 | Lifetime 2 |
|---|---|---|---|---|---|---|
| BST-NTs $B_1$ | / | / | / | 0-15 ps | Double Exp. | 0.92 ± 0.07 ps & 8.96 ± 0.56 ps |
| BST-NSs $B_2$ | / | / | / | 0-15 ps | Double Exp. | 0.56 ± 0.02 ps & 2.71 ± 0.23 ps |
| BST-MHs $B_1$ | 0-0.8 ps | Single Exp. | 0.26 ± 0.02 ps | 0.8-15 ps | Double Exp. | 0.61 ± 0.03 ps & 3.61 ± 0.23 ps |
| BST-MHs $B_2$ | 0-0.8 ps | Single Exp. | 0.21 ± 0.05 ps | 0.8-15 ps | Double Exp. | 0.93 ± 0.07 ps & 15.3 ± 2.5 ps |

**Supplementary Table 3 Comparison of the NIR activity of our catalyst with other catalysts for PEC hydrogen production.**

| Materials | Solution | Wavelength | NIR Photocurrent | IPCE | Stability | Ref. |
|---|---|---|---|---|---|---|
| **BST/CoSeTe-1%** | 0.08 M $Na_2S$ 0.50 M $Na_2SO_3$ | 800-1100 nm | 1.5 mA $cm^{-2}$ (0.6 $V_{RHE}$) | 36% at 800 nm (0.6 $V_{RHE}$) | 120h | This work |
| **$Fe_2O_3$** | 1 M NaOH | 830 nm | 2 μA $cm^{-2}$ (1.4$V_{RHE}$) | NA | NA | 2 |
| **($NaYF_4$:Yb-Tm)/($Cu_2O$)** | 0.1 M $Na_2SO_4$ | Xenon lamp (500 W) with 850 nm cutoff filter | 4 μA $cm^{-2}$ (0.3 $V_{Ag/AgCl}$) | NA | NA | 3 |
| **$Ag_2O/TiO_2$** | 1 M $Na_2SO_4$ | >784 nm 1300 mW $cm^{-2}$ | 20 μA $cm^{-2}$ (0.4 $V_{SCE}$) | 0.49% | NA | 4 |
| **($Y_2O_3$:Yb–Er)/$Bi_2S_3$** | 0.5 M $Na_2SO_4$ | 980 nm (1W) | 12 μA $cm^{-2}$ (0.3 $V_{Ag/AgCl}$) | NA | NA | 5 |
| **$In_2TiO_5$** | 1 M $Na_2SO_4$ | Xenon lamp (300 W) with 800 nm cutoff filter | 0.5 μA $cm^{-2}$ (1.0 $V_{Ag/AgCl}$) | NA | 3000 s | 6 |
| **$WO_{3}$-x-HT** | 1 M $Na_2SO_4$ | 780-2000 nm | 0.1 mA $cm^{-2}$ (1.0 $V_{Ag/AgCl}$) | NA | NA | 7 |
| **N-C-$TiO_2$ NRs** | 0.1 M $Na_2SO_4$ | Xenon lamp (300 W) with 800 nm cutoff filter | 35 μA $cm^{-2}$ (1.0 $V_{RHE}$) | NA | NA | 8 |
| **$C_3N_4$** | 0.5 M $Na_2SO_4$ | Red Light | 1.5 μA $cm^{-2}$ (0.8 $V_{Ag/AgCl}$) | NA | NA | 9 |
| **$FeS_2/TiO_2$** | 0.24 M $Na_2S$ 0.35 M $Na_2SO_3$ | 808nm laser 100mW $cm^{-2}$ | 1.9 mA $cm^{-2}$ (0.1 $V_{Ag/AgCl}$) | 3% at 808 nm 300 mW·$cm^{-2}$ | 2 h | 10 |
| **NP-NR-$BiVO_4$** | 0.1 M PBS (pH=7) | 800-2000 nm 363mW $cm^{-2}$ | 5 μA $cm^{-2}$ (0.4 $V_{Ag/AgCl}$) | NA | NA | 11 |
| **$NaYF_4$:$Yb^{3+}$, $Er^{3+}$/Au/CdS** | 0.2 M $Na_2SO_4$ | Xenon lamp (300 W) with 700 nm cutoff filter | 6 nA $cm^{-2}$ | NA | NA | 12 |
| **Defect-engineered $TiO_2$ nanotube** | PBS (pH=7.4) | 778nm laser 20mW $cm^{-2}$ | 6 μA $cm^{-2}$ (0 $V_{Ag/AgCl}$) | 0.22% at 800nm without external bias | NA | 13 |
| **Au@Nb@HxK1-x$NbO_3$ nanopeapods** | 0.5 M $Na_2SO_4$ | AM 1.5G, 100 mW $cm^{-2}$ with 780 nm cut-off filter | 0.1 μA $cm^{-2}$ (1 $V_{Ag/AgCl}$) | < 0.1% at 800nm (1 $V_{Ag/AgCl}$) | NA | 14 |
| **Au clusters/$TiO_2$** | 0.1 M LiI and 0.1 M tetra-n-butylammonium perchlorate | 460–800 nm light (75 mW $cm^{-2}$) | 450 μA $cm^{-2}$ (0.5 $V_{SHE}$) | 0.8% at 800 nm | 200 s | 15 |
| **$Ag_2Se$@$Ag_2S$ core–shell NCs** | 0.5 M $Na_2S$, 2.0 M S | 940 nm (10 mW $cm^{-2}$) | 2.2 μA $cm^{-2}$ | NA | 6 h | 16 |

| | | | | | | |
|---|---|---|---|---|---|---|
| **Au/$TiO_2$ nanocavity** | Tris-HCl buffer solution (pH 7.4) | NIR light | 4.5 µA $cm^{-2}$ (0 $V_{Ag/AgCl}$) | 0.15% at 800nm (0 $V_{Ag/AgCl}$) | NA | 17 |
| **$TiO_2$/BTO/ $Ag_2O$** | 1 M NaOH | 250 W infrared lamp | 4.0 µA $cm^{-2}$ (0.3 $V_{Ag/AgCl}$) | 0.5% at 800nm (0.5 $V_{Ag/AgCl}$) | 1.5 h | 18 |
| **Si NWs** | 0.24 M $Na_2S$ 0.35 M $Na_2SO_3$ | Simulated sunlight illumination (10 mW $cm^{-2}$) | 0.12 mA $cm^{-2}$ (1.2 $V_{RHE}$) | 12% at 800nm (0.88 $V_{RHE}$) | NA | 19 |
| **Si NWs** | 0.25 M KOH | AM 1.5G 100 mW $cm^{-2}$ | 6.4 µA $cm^{-2}$ (1.2 $V_{RHE}$) | 8% at 800nm (2.5 $V_{RHE}$) | 650 s | 20 |
| **n-Si** | 1 M NaOH | AM 1.5G 100 mW $cm^{-2}$ | NA | NA | 0.5 h | 21 |

**Supplementary Table 4 Fitting results of electrochemical impedance spectroscopy.**

| | $R_s$/Ω | $R_1$/Ω | $CPE_1$/F | $R_2$/Ω | $CPE_2$/F |
|---|---|---|---|---|---|
| **BST-MHs** | 35.73 | 15.69 | $9E^{-5}$ | 593.1 | $6.7E^{-5}$ |
| **BST-NTs** | 40.5 | 50.6 | $1.38E^{-5}$ | 868.5 | $4.43E^{-5}$ |
| **BST-NSs** | 34.7 | 48.7 | $1.6E^{-5}$ | 1622 | $4.26E^{-5}$ |

To quantify the changes in the transport impedance of the BST material, we fitted the impedance spectra using Zview (Supplementary Table 4). The fitting results show that *R*1 and *R*2 of BST-MHs are 15.69 Ω and 593.1 Ω, respectively, and *CPE*1 and *CPE*2: 9.0 x $10^{-5}$ F and 6.7 x $10^{-5}$ F, respectively. In comparison, the *R*1 and *R*2 of BST-NTs and BST-NSs samples are 50.6 Ω and 868.5 Ω, 48.7 Ω and 1622 Ω, respectively, and *CPE*1 and *CPE*2 are 1.38 x $10^{-5}$ F and 4.43 x $10^{-5}$ F, 1.6 x $10^{-5}$ F and 4.26 x $10^{-5}$ F, respectively. This indicates that the bulk and surface transmission impedances of BST-MHs are smaller than those of BST-NTs and BST-NSs samples.

The decrease in transmission impedance proves that the formation of a lattice-matched heterojunction is conducive to the migration of carriers from the bulk to the surface — greatly mitigating carrier recombination in the bulk phase, and increasing the number of surface-reaching carriers.

**Reference**


1. Maiolo, J. R. *et al.*, Macroporous Silicon as a Model for Silicon Wire Array Solar Cells. *J. Phys. Chem. C* **112**, 6194-6201 (2008).
2. Du, C., Zhang, M., Jang, J-W, Liu, Y., Liu, G-Y, Wang, D., Observation and Alteration of Surface States of Hematite Photoelectrodes. *J. Phys. Chem. C* **118**, 17054-17059 (2014).
3. Jia, H. *et al.*, Near-Infrared Light-Induced Photocurrent from a ($NaYF_4$:Yb-Tm)/($Cu_2O$) Composite Thin Film. *Adv. Energy Mater* **5**, 1401041 (2015).
4. Li, C., Hsieh, J. H., Cheng, J. C., Huang, C. C., Optical and photoelectrochemical studies on $Ag_2O/TiO_2$ double-layer thin films. *Thin Solid Films* **570**, 436-444 (2014).
5. Jia, H., *et al.*, Fabrication of the ($Y_2O_3$:Yb-Er)/$Bi_2S_3$ composite film for near-infrared photoresponse. *J. Mater. Chem. A* **3**, 5917-5922 (2015).
6. Wei, D. *et al.*, Band Gap Engineering of $In_2TiO_5$ for $H_2$ Production under Near-infrared Light. *ACS Appl. Mater. Interfaces* **7**, 20761-20768 (2015).
7. Yan, J. *et al.*, Tungsten Oxide Single Crystal Nanosheets for Enhanced Multichannel Solar Light Harvesting. *Adv. Mater* **27**, 1580-1586 (2015).
8. Hu, Z., Yu, J. C., Ming, T., Wang, J., A wide-spectrum-responsive $TiO_2$ photoanode for photoelectrochemical cells. *Appl. Catal. B* **168**, 483-489 (2015).
9. Tiwari, J. N. *et al.*, Accelerated Bone Regeneration by Two-Photon Photoactivated Carbon Nitride Nanosheets. *ACS Nano* **11**, 742-751 (2017).
10. Wang, D. Y. *et al.* Iron Pyrite/Titanium Dioxide Photoanode for Extended Near Infrared Light Harvesting in a Photoelectrochemical Cell. *Sci. Rep.* **6**, 20397 (2016).
11. Yang, W. *et al.*, Plasmonic Pd Nanoparticle- and Plasmonic Pd Nanorod-Decorated $BiVO_4$ Electrodes with Enhanced Photoelectrochemical Water Splitting Efficiency Across Visible-NIR Region. *Nanoscale Res. Lett* **11**, 283 (2016).
12. Feng, W. *et al.*, Near-infrared-activated $NaYF_4$:$Yb^{3+}$, $Er^{3+}$/Au/CdS for $H_2$ production via photoreforming of bio-ethanol: plasmonic Au as light nanoantenna,

energy relay, electron sink and co-catalyst. *J. Mater. Chem. A* **5**, 10311-10320 (2017).

13. Wu, W., Zhang, Z., Defect-engineered $TiO_2$ nanotube photonic crystals for the fabrication of near-infrared photoelectrochemical sensor. *J. Mater. Chem. B* **5**, 4883-4889 (2017).
14. Chen, Y. C., Hsu, Y. K., Popescu, R., Gerthsen, D., Lin, Y. G., Feldmann, C., $Au@Nb@H_xK_{1-x}NbO_3$ nanopeapods with near-infrared active plasmonic hot-electron injection for water splitting. *Nat. Commun.* **9**, 232 (2018).
15. Kogo, A., Sakai, N., Tatsuma, T., Photoelectrochemical analysis of size-dependent electronic structures of gold clusters supported on $TiO_2$. *Nanoscale* **4**, 4217-4221 (2012).
16. Tang, S. et al., Precursor reactivity differentiation for single-step preparation of $Ag_2Se@Ag_2S$ core-shell nanocrystals with distinct absorption and emission properties enabling sensitive near-infrared photodetection. *J. Mater. Sci.* **53**, 11355-11366 (2018).
17. Li, Z., Zhou, X., Yang, J., Fu, B., Zhang, Z., Near-Infrared-Responsive Photoelectrochemical Aptasensing Platform Based on Plasmonic Nanoparticle-Decorated Two-Dimensional Photonic Crystals. *ACS Appl. Mater. Interfaces* **11**, 21417-21423 (2019).
18. Liu, Z. et al., Piezoelectric-Effect-Enhanced Full-Spectrum Photoelectrocatalysis in p-n Heterojunction. *Adv. Funct. Mater.* **29**, 1807279 (2019).
19. Liu, Q., Wu, F. L., Cao, F. R., Chen, L., Xie, X. J., Wang, W. C., Tian, W., Li, L., A Multijunction of $ZnIn_2S_4$ Nanosheet/$TiO_2$ film/Si Nanowire for Significant Performance Enhancement of Water Splitting. *Nano Res.* **8**, 3524-3534 (2015).
20. Wu, F. L., Liao, Q. L., Cao, F. R., Li, L., Zhang, Y., Non-noble Bimetallic $NiMoO_4$ Nanosheets Integrated Si Photoanodes for Highly Efficient and Stable Solar Water Splitting. *Nano Energy* **34**, 8-14 (2017).
21. Chen, Y. W. et al., Atomic layer-deposited tunnel oxide stabilizes silicon photoanodes for water oxidation. *Nat. Mater.* **10**, 539-544 (2011).

.
