# Supplementary material for: Boosting photoelectrochemical efficiency by near-infrared-active lattice-matched morphological heterojunctions
Source: Nat Commun. 2021 Jul 14;12:4296. doi: 10.1038/s41467-021-24569-9 (PMC8280183; doi:10.1038/s41467-021-24569-9)
Supplement: Supplementary file 2 — Description of Additional Supplementary Files [file 41467_2021_24569_MOESM2_ESM.pdf]

Description of Additional Supplementary Files

Supplementary Movie 1

The PEC H_2 production performance of the BST-MH photoanode under visible light illumination (λ >420 nm, 100 mW cm^{-2})
